# Crowdsourced and AI-generated age-of-acquisition (AoA) norms for vocabulary in print: Extending the Kuperman et al. (2012) norms

**DOI:** 10.3758/s13428-025-02843-8

**Published:** 2025-10-06

**Authors:** Clarence Green, Anthony Pak-Hin Kong, Marc Brysbaert, Kathleen Keogh

**Affiliations:** 1 Faculty of Education, University of Hong Kong, Pok Fulam, Hong Kong; 2 Department of Experimental Psychology, Ghent University, Gent, Belgium; 3 Institute of Innovation, Science and Sustainability, Federation University, Mount Helen, Australia

**Keywords:** Age of acquisition, Large language model, AI, Word norms, Crowdsourcing, Vocabulary

## Abstract

This paper revisits the age-of-acquisition (AoA) norms of Kuperman et al. (2012). Three studies were conducted. Study 1 reports a crowdsourcing ‘megastudy’ obtaining 790,024 estimates from participants with the age they could first read and write 11,074 early acquired words from Kuperman et al. (2012). The study aimed to differentiate between oral language receptive AoA and print-based AoA. The results correlate well with the original estimates, offering, as hypothesized, higher AoAs for reading/writing. These are released as supplements to the original norms. Study 2 explored the potential of large language models (LLMs), specifically GPT-4o, to replicate these crowdsourced AoA estimates. The findings indicated a strong correlation between AI-generated estimates and human judgments, showing the utility of AI in estimating AoA and developing norms for psycholinguistic and educational research in lieu of crowdsourcing. Study 3 leveraged AI to extend estimates to all well-known words in Kuperman et al. (2012) and the English Crowdsourcing Project (ECP). Study 3 also investigated a trained model fine-tuned on 2000 ratings from Kuperman et al. (2012). Fine-tuning increased alignment with human ratings, though comparisons with untrained models suggested that fine-tuning is not essential in English for obtaining useful AoA estimates. Both trained and untrained AI-generated norms correlated highly with human ratings and performed well in accounting for word processing times and accuracy in regressions. Uses and limitations of the AI estimates are discussed. All resources are made available in the Open Science Framework and can be used freely for research and education.

## Introduction

The age of acquisition (AoA) of a word refers to the approximate age when a word was first learned and is one of the most widely used variables in the behavioral sciences (Kuperman et al., 2012; Smolik & Filip, 2022; Brysbaert, 2017). For the bulk of the everyday vocabulary known by native speakers and early onset multilinguals, the AoA usually ranges from childhood to adolescence (Nation & Coxhead, 2021), though words continue to be learned throughout life (Brysbaert et al., 2016; Green et al., 2024a, b). AoA is one of the “big five” variables for researchers working with word stimuli (Brysbaert et al. 2019), alongside word frequency, word length, similarity to other words, and word prevalence (how many people know the word in the population) (Mandera et al., 2020). These variables account for more variance in word processing than other lexical characteristics (Kuperman et al., 2012; Elsherif et al., 2023).

AoA norms have typically been developed by presenting a target word to a pool of participants and asking for their estimates of when they first understood the word (see below for other approaches). Researchers then compute the average estimated age across respondents. The most influential AoA norms in English are those developed by Kuperman, Stadthagen-Gonzalez and Brysbaert (2012), containing approximately 31,000 words.

A limitation of the Kuperman et al. (2012) norms is that they focused on receptive comprehension. Participants were instructed to estimate the age at which they first understood a word, regardless of whether they could read, write, or use it productively in speech. For educational research, it is also useful to have information on AoA for reading and writing. In the early years, children acquire words through oral language comprehension before print (Hoover & Tunmer, 2020), whereas after a few years of education, most new words are acquired through reading (Share, 1995). For early acquired vocabulary, the AoA with regard to print is probably higher than the estimates in Kuperman et al. (2012), though likely converges for words with higher values (i.e., the older AoAs are more likely to have been print-acquired).

This paper reports three related studies. Study 1 consists of a crowdsourcing ‘megastudy’ (Cortese, 2019), which obtained 790,024 estimates from Amazon Mechanical Turk of when participants could first read and write 11,074 early acquired words from Kuperman et al. (2012). We focused on words from the original database with AoAs under the age of 10. Although this is an arbitrary threshold, words below age 10 in Kuperman et al. (2012) would contain the most likely candidates benefiting from disambiguation between oral language (receptive) and print acquisition. The new print-based AoA crowdsourced norms are validated and released to extend Kuperman et al. (2012).

Study 2 examined whether AoA estimates from large language models (LLMs) could replicate the crowdsourced data from Study 1. We investigated AI-generated estimates from GPT-4o (https://openai.com/). Recent research has suggested LLMs are surprisingly good at estimating word features (Brysbaert et al., 2025; Martínez et al., 2025a; Trott, 2024). Study 2 is an ideal test case of how well AI correlates with human judgements because: a) zero-shot prompting is used, meaning that values are obtained from conversational prompting without intentionally fine-tuning the AI with human training data; b) the crowdsourced data from Study 1 were not yet released at the time of testing, ruling out that the AI incorporated the data into web-crawled training materials, a problem known as data leakage/contamination.

Study 3 extended the approach of Study 2 to obtain further AoA norms via AI. It generated AoA estimates for all words in the English Crowdsourcing Project (ECP) (Brysbaert et al., 2019; Mandera et al., 2020) known by more than 95% of people, many without AoA estimates in Kuperman et al. (2012). Additionally, Study 3 investigated the effect of model fine-tuning on estimates of AoA compared to the zero-shot method. Sendín et al. (2025) recently found that AI-generated AoAs for Spanish were substantially improved through training the LLM on 2000 crowdsourced estimates. We therefore explored this for English, using 2000 samples from Kuperman et al. (2012). We also leveraged this model for possible print AoAs for words in Kuperman et al. (2012) above the age range of Study 1. All new crowdsourced and AI-generated resources are provided in the OSF (https://tinyurl.com/3ck778nr).

This research makes two main contributions. Firstly, new crowdsourced and AI resources are provided for researchers. Secondly, the studies collectively show that LLMs provide good approximations of AoA norms, thus LLMs (trained and untrained) can be used to provide researchers with initial estimates of words for which no human data is yet available.

## Age-of-acquisition norms: Previous approaches

The predominant research method over the past 10–15 years for developing large-scale AoA norms has been online ‘crowdsourcing’. This involves a large number of adult participants subjectively estimating the age at which they think they learned a word. The Kuperman et al. (2012) norms represent the benchmark for AoA values and have been the most influential. These were obtained through Amazon Mechanical Turk (AMT), a technological innovation at the time. AMT is an online platform connecting researchers and participants, providing access to a large number of ‘workers’ who complete HITs (Human Intelligent Tasks; Difallah et al., 2018).

Kuperman et al. (2012) designed a HIT to collect AoAs that prompted workers to consider a target word and type the estimated age when they “first understood that word if somebody had used it in front of them, EVEN IF THEY DID NOT use, read, or write it at the time” (Kuperman et al., 2012, p. 980). Note the emphasis in capital letters: the prompt tapped oral language receptive understanding. Kuperman et al. (2012) crowdsourced 696,048 estimates from 1729 workers over 6 weeks. The norms contain averaged estimates for 30,121 lemmas (nouns, verbs, and adjectives), based on at least 18 estimates.

Kuperman et al. (2012) correlated estimates with lab-collected norms from previous studies, finding correlations of *r* =.93 with 2544 words from Cortese and Khanna (2008), *r* = 0.83 with 1787 words from Bird et al. (2001), and *r* =.86 with 3117 words from Stadthagen-Gonzalez and Davis (2006). These data indicated convergent validity and also suggested that AoA values differ little one decade to the next. The AoA norms were investigated as word processing predictors. They correlated strongly with (log) word frequency (*r* = –.685), standardized response times (*r* =.67) and accuracy (*r* = – 507) in the English Lexicon Project/ELP (Balota et al., 2007). Stepwise multiple regression indicated that the estimates explained substantial variance in lexical decision accuracy and speed, after the effects of word frequency and word length were accounted for.

Another example of crowdsourcing is the Glasgow norms (Scott et al., 2019). These contain averaged AoA estimates for 5553 English words (along with ratings for nine other psycholinguistic variables not pertinent here). Methodologically, the Glasgow norms differ from Kuperman et al. (2012) by collecting estimates on a seven-point Likert scale, with each interval representing 2-year periods (0–12 years) and the final scale point representing a 13+ age range. Participants were 829 university students on an online platform at the host university who rated lists of words containing either 101 or 150 items. Stimuli were content words (nouns, verbs, adjectives, adverbs). Correlations with previously released estimates validated the norms: ranging from *r* =.89 (Kuperman et al., 2012), *r* =.86 (Bird et al., 2001) up to *r* =.95 (Davies et al., 2016), though with an outlier at *r* =.20 (Khanna & Cortese, 2011).

Direct testing is an alternative way to estimate AoA. Here, researchers investigate at which age children can recognize and produce words. Given the costs and resources involved in direct testing, the number of words is usually limited to a few hundred. An exception is Dale and O’Rourke (1981), who tested children in US schools over decades starting in the 1960 s, and obtained estimates of the grades at which 44,000 words were understood. The researchers visually presented words to children, who were asked to select from amongst four possibilities the correct meaning. A word was assigned a grade level when 67–80% of a sample of 200 or more children knew the meaning. Grades tested included 4, 6, 8, 10, and 12 (as well as some college students). Dale and O’Rourke (1981) reported, amongst other contributions, that approximately 30,000 words were known by at least 67% of students from Grades 4–12.

Brysbaert and Biemiller (2017) validated the Dale and O’Rourke (1981) norms as an alternative to crowdsourced AoAs by correlating the data with Kuperman et al. (2012). Further, they showed that these estimates contributed to word processing times when regressed onto the ELP norms (Balota et al., 2007), though not as strongly as crowdsourced data. Brysbaert and Biemiller (2017) report a correlation of *r* =.757 with the Kuperman et al. (2012) AoA norms and an *R*^2^ =.54 on RT in the ELP in a simple model of LDT = frequency + length + AoA. The value of these norms as augment to Kuperman et al. (2012) is that they offer estimates for more words not in other databases and also estimates for polysemous words.

A third way to collect large-scale AoA norms involves corpus analysis (Korochkina et al., 2024), and natural language processing (NLP). Botarleanu et al. (2022), for instance, estimated predicted age of exposure (AoE) for 44,000 words by using semantic vectors (Word2vec embeddings) trained on age-graded and cumulatively increasing text corpora. Using multiple regression, the vectors were translated into predicted AoA values by aligning the word2vec vector spaces with different ages. Extending this approach, Flor et al. (2024) analyzed and categorized 22,000 words from 74 instructional vocabulary lists used at different grades in USA schools, deriving the Vocabulary eXpected Grade Levels (VXGL) for 126 thousand English words. The VxGL values correlated well (*r* =.78) with Brysbaert and Biemiller (2017).

## Augmentation of psycholinguistic norms with AI

In the past 2 years, several researchers observed that large language models (LLMs) provide information about word features that closely resemble human information. LLMs are machine learning systems trained on vast amounts of text, enabling a probabilistic ability to predict the next word (Hussain et al., 2024). Such models and the interfaces built around them allow users to collect language-related information in the same way as with human participants.

For instance, Brysbaert et al. (2025) recently derived familiarity estimates for more than 400,000 words and multi-word expressions/MWEs from GPT-4o. Subjective familiarity is a participant’s estimate of how familiar they are with a word, typically obtained via a Likert scale (1–7). Brysbaert et al. (2025) asked GPT-4o to provide Likert ratings of familiarity using a conversational prompt that explained familiarity similar to what human participants would receive in crowdsourced data collection (i.e., the extent to which the target word is easily recognizable and seen/heard often). The AI estimates correlated well with human ratings from.72 to.87 (non-linear) and.66 to.86 (linear). These were in the same range as correlations amongst human datasets. The authors also found that AI-familiarity norms correlated with AoA norms at –.67 (Kuperman et al., 2012), suggesting that the words acquired early are more familiar.

Martínez et al. (2025a) collected AI-generated norms for concreteness, valence, and arousal for 63,680 MWEs and 126,397 individual English words. They had GPT-4o rate the concreteness of the input on a scale of 1 (least abstract) to 5 (most abstract) and provided examples in their prompt of what would be rated a 5. The LLM returned estimates that correlated *r* =.8 with human ratings. Norms for valence were collected by asking, on a scale of 1–9, for ratings of how the word/MWE made people feel (9 = very positive and good; 1 extremely negative). These correlated with human judgments (from.79 to.93). Arousal norms were collected and similarly correlated with human benchmarks from.56 to.85.

AoA was investigated by Trott (2024). He provided an LLM with the same instructions as the Glasgow norms, though asked for an estimated age rather than a rating along a Likert 1–7. Trott (2024) reported a correlation of 0.72 between the AI-generated estimates and the Glasgow ratings. While the data of Trott (2024), Brysbaert et al. (2025) and Martínez et al. (2025a) demonstrate that AI can produce valid estimates for a range of word characteristics, as Trott (2024) states “an estimate of the reliability of LLM-generated norms depends upon a human “gold standard” with which to evaluate those norms-which is a key argument for keeping humans in the loop” (p. 6097). Further, he noted the concern of ‘contamination’, referring to the potential that LLMs include norms available on the web in their training data. Similar concerns were raised by Brysbaert et al. (2025) and Martínez et al. (2025a).

Sendín et al. (2025) recently reported that a fine-tuned/trained Spanish LLM model can provide AoA estimates even closer to human ratings than a zero-shot/untrained model. Sendín et al. (2025) initially obtained a correlation of *r* = 0.75 between human AoA ratings from five different Spanish studies and zero-shot GPT-4o AoA estimates. The AoA ratings from the different human studies correlated with each other at *r* = 0.85. Sendín et al. (2025) then fine-tuned a GPT-4o model with 2000 words from crowdsourced ratings. Based on the deviation between the AI estimate and the human ratings as feedback, the model’s connection weights were updated to better align with human ratings. Sendín et al. (2025) found that the fine-tuned AoA estimates increased to *r* = 0.85 with the human ratings, bringing the model in line with the human rating studies.

## Study 1: Crowdsourced AoA norms for print

Building on the foregoing research, the goal of Study 1 was to extend the Kuperman et al. (2012) norms with estimates representing print through a new crowdsourcing megastudy. As noted, there are different methods for collecting AoA norms, which may tap different aspects of word knowledge: for extensive reviews, see Brysbaert (2017) and Elsherif et al. (2023). Human ratings are arguably the most informative and feasible, though there is concern they may be influenced by non-age-related effects such as experience and word familiarity (Brysbaert & Biemiller, 2017). Also, some discrepancies have been documented, such as mismatches between the AoA norms for some words and children’s use of these words in speech (Smolik & Filip, 2022). Crowdsourced AoA norms nonetheless provide human data that correlate highly with other methods that have been tried (Brysbaert, 2017) and provide human data that can be used to test and train AI.

We were particularly interested in the extent to which the Kuperman et al. (2012) norms were valid for reading and writing AoA. We hypothesized that the print AoA would be higher, on average, than the Kuperman et al. (2012) norms for earlier acquired words.

### Method

We followed the procedure used by Kuperman et al. (2012). For reference, in their methods, each stimuli list in AMT contained 362 words, made up of 300 test words, 52 controls, and ten practice words. Their quality checks included that the AoA estimates had to correlate above *r* =.20 with the Bristol norms (i.e., 52 controls). Cleaning involved removing empty cells (approx. 7%), estimates beyond self-reported age or unknown (approx. 1% of data), and any participants if their estimates did not correlate with the Bristol norms at *r* = 0.4 (15% of the data). They also removed AoAs above 25 (approx. 1%).

#### Stimuli

Target stimuli were 11,074 lemmas in Kuperman et al. (2012) with AoA estimates under age 10. This age span approximately covers the elementary school years when children acquire literacy and differences amongst oral language, reading, and writing AoA for words would be more pronounced (Share, 1995).

#### Data collection

In AMT, participants were presented with approximately 110 words. Target words from Kuperman et al. (2012) were first ordered from youngest age to oldest, then one word from every 100 sampled and placed in a list. Thus, participants saw words from different estimated AoA ranges. This prevented them from seeing a wordlist containing words with similar AoAs.

Participant location was restricted to the USA, workers were required to have a 98% prior approval rating, and an age of initial English learning of under 5 years old. Completion time was set to Pacific Standard Time 5 a.m. to 23:59 p.m. so as to improve data quality (i.e., attention to task) and minimize data coming from outside the US via VPNs. Participants provided consent after a Plain Language Information Sheet (PLIS) and were paid $1 USD for usable data. Ethics approval was obtained from the host institution (HREC Reference Number: EA240234). Participants were asked to estimate AoA as follows: “Reading: When you are estimating your reading acquisition of a word, we mean the age at which you would have understood that word if you read it in print. Writing: When you are estimating written acquisition age, what we mean is when you estimate you could spell the word correctly”.

Full instructions, AMT code, and information sheets are provided in the OSF (https://tinyurl.com/3ck778nr). Participants were given context that included explaining that some words may have been understood at an earlier age in speech but only later able to be read or written. Participants were instructed that if they did not know a word, or learned the word in their adult life, they could select ‘Not Applicable’. They responded on a slider with an upper scale of 20, as in (1).

(1) Mechanical Turk data collection instrument
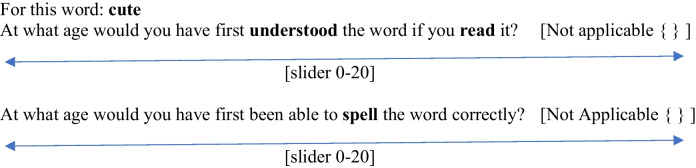


The slider showed the numerical value indicated (in years), so that participants knew what value they reported. An alternative would have been to ask participants to enter numbers by typing them, as an age or Likert value. However, the slider was thought to reduce typing error. Participants estimated reading first, followed by writing. There is therefore the potential for order effects (i.e., the writing estimate influenced by the initial reading estimate). Participants were able to complete multiple lists. Data that did not correlate with the Kuperman et al. (2012) norms at.25 or above were rejected. The rejection rate was high (over 50%), atypical for psycholinguistic research in the lab, but consistent with online AMT data collection, where robust quality checks are needed (Patel et al., 2024). The average number of valid unique ratings collected for each word was 35.61.

#### Data cleaning

Several forms of the primary data are available in the OSF for other researchers. The first is the raw data. The second is a version with cleaner formatting and values age 3 and below removed, since reading/writing words before age 3 is uncommon so these are likely low-quality estimates. Most children begin to read words about age 4 (Horowitz‐Kraus et al., 2017), so this threshold seemed reasonable (lowest print value in this version is 3.5). The third version additionally replaced outliers for each word with the average rating for that word and this is the data used in the following analyses. Outliers were defined as 2.5 standard deviations from the mean. Analyses can be performed using different thresholds and cleaning, if desired, using the raw data in the OSF.

### Results

#### Participant statistics

We crowdsourced 790,024 valid estimates: 388,568 for reading and 401,860 for writing. Estimates came from 3826 participant submissions, provided by 1351 unique workers. Demographic data is reported in Table 1.

**Table 1:** Demographics

	Participant submissions	Unique participants	Age (mean)	Reading AoA (mean)	Writing AoA (mean)
Male	2251	897	37.02	8.45	9.07
Female	1575	509	37.95	7.99	8.67

Demographic data is unbalanced, so group differences need to be cautiously interpreted. Nonetheless, Table 1 bears comparison to Kuperman et al. (2012). They had more than twice the number of female-to-male participants (1136 vs. 593, respectively), whereas the current study has more males. In the original, females gave slightly higher estimates, while in the current study, males gave higher estimates. This could suggest that when a larger sample is taken, crowdsourced data reveal findings that females tend to acquire early vocabulary slightly earlier than males (Huttenlocher et al., 1991). Table 1 indicates that the average age of participants was 37–38 years, and similar for both genders. Kuperman et al. (2012) found a weak (*r* = 0.07) but significant relationship between age and AoA. The current study confirms a similar negligible relationship in the opposite direction (*r* = –.084, *p* <.001). Participant age does not seem to strongly affect AoA ratings. Table 2 reports participant data by education level.

**Table 2:** Reported education level and AoA estimates

	*N*	AoA reading (mean)	AoA writing (mean)
High school diploma	34	10.51	11.21
Associates degree	90	9.21	11.81
Bachelor’s degree	2832	8.23	8.78
Graduate degree	786	8.28	9.13

As with Kuperman et al. (2012), most participants had bachelor’s degrees. The original study found a slight trend toward more highly educated individuals providing earlier AoA estimates. A similar trend is in the current data. Those with a high school diploma gave later AoA estimates, followed by participants with associate degrees, then those reporting university-level degrees (bachelor/graduate). The small sample for high school diploma and associate degree holders prevents strong conclusions, but the results may reflect that those who continue to higher levels of education were faster to learn to read and write words in childhood. Alternatively, participants with higher education may estimate earlier vocabulary knowledge to fit with self-perceptions of having gone further in education.

#### AoA descriptive statistics and comparison with Kuperman et al. (2012)

Table 3 reports the AoA descriptive statistics from the crowdsourced data compared with Kuperman et al. (2012) for early acquired words.

**Table 3:** Means, standard deviations, and range

Descriptives	Mean	Std. Dev.	Min.	Max.
AoA Kuperman et al. (2012)	7.70	1.69	1.58	9.97
AoA Reading	8.20	1.28	4.19	12.55
AoA Written	8.88	1.32	4.49	12.58

**Fig. 1:**
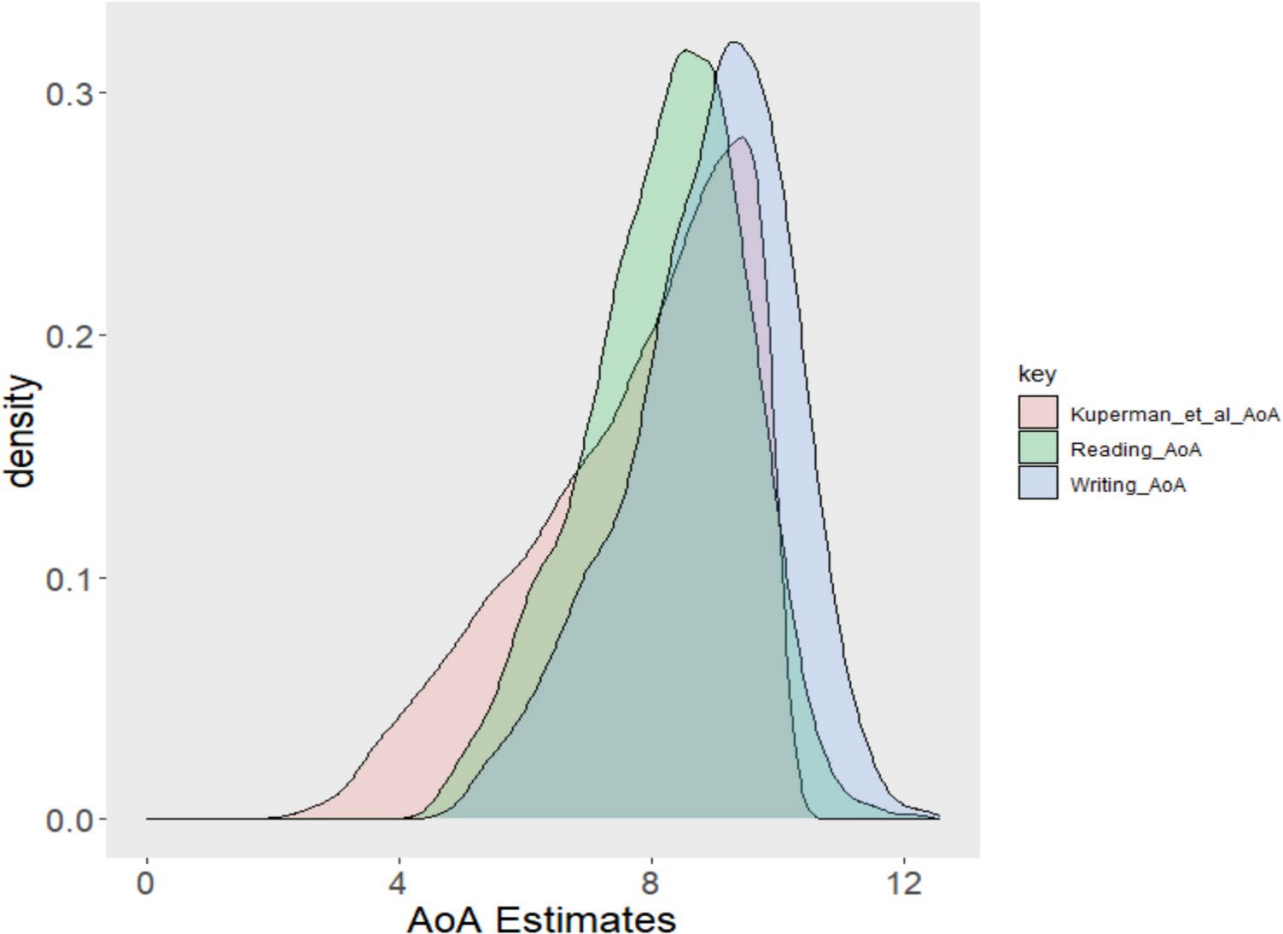
Density plot of the crowdsourced AoA ratings

The hypothesis that AoAs for print would, on average, be higher than the Kuperman et al. (2012) norms is confirmed in Table 3. The Kuperman et al. (2012) norms are the lowest, followed by reading, then writing, which makes intuitive sense. The Kuperman et al. (2012) norms range from 1.58 to 9.97 years old, while the new print norms range from table4.19 for reading and 4.49 for writing up to 12.55 and 12.58, respectively. Figure 1 shows density plots for the print and Kuperman et al. (2012) ratings.

**Fig. 2:**
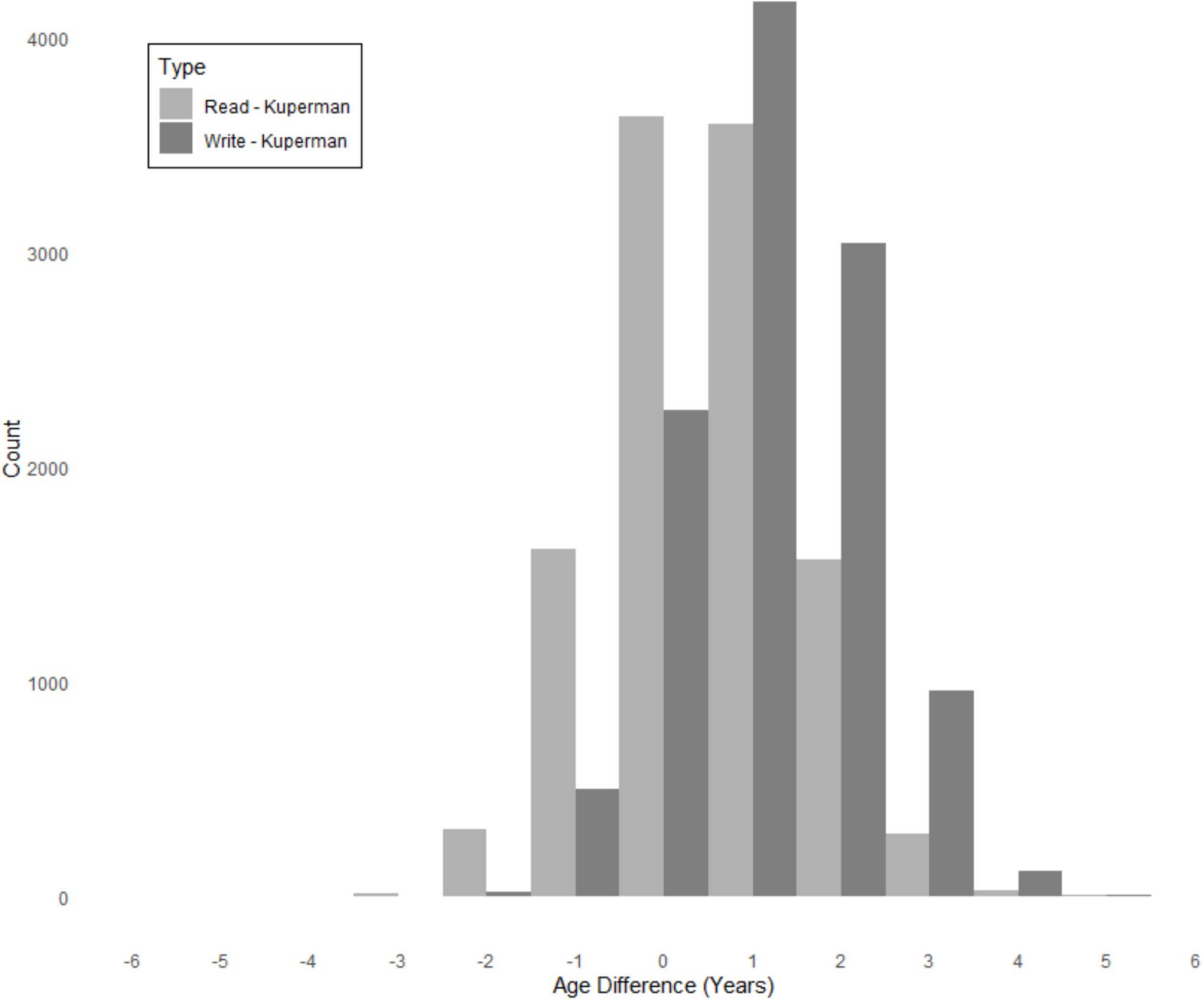
Distribution of rounded age differences for the AoA ratings

The plots indicate that the estimates are symmetric and largely overlap, with a higher density around ages 8 for reading and 9 for writing compared to Kuperman et al. (2012). The distribution of rounded age differences between the reading and writing estimates and the Kuperman et al. (2012) estimates is reported in Fig. 2

The reading and writing estimates are mostly between 1 and 2 years higher than Kuperman et al. (2012). The reading estimates are closer to the original estimates, while more of the writing estimates have greater age differences. For example, 1083 of the estimates for writing have ages greater than 3 years, their Kuperman et al. (2012) values, though only 347 do for reading. Interestingly, some words have been estimated as acquired in print earlier than the previous norms. These mismatches include words such as ‘refuse’ (read: 6.9; write: 7.6; Kuperman et al: 9.3); hourly (read: 7.1; write: 8.1; Kuperman et al., 9.5), ‘reject’ (read: 6.7; write: 7.6; Kuperman et al., 9.5) and ‘bro’ (read: 6.6; write: 7.4; Kuperman et al., 9.2).

Table 4 shows further examples of words with substantially different print AoAs than Kuperman et al. (2012), as well as words with largely similar estimates.

**Table 4:** Examples of words with different and similar print AoAs cf. Kuperman et al. (2012)

Reading				Writing			
Substantially different estimated AoAs							
	AoA Read	Kuperman et al. (2012)	Age diff.		AoA Write	Kuperman et al. (2012)	Age diff.
Mother	7.3	2.6	4.7	Spaghetti	8.8	4.3	4.5
Sleigh	10.1	6.0	4.1	Ballerina	9.3	5.3	4.0
Potty	6.0	2.3	3.8	Impatient	11.4	7.4	4.0
Yucky	8.1	4.2	3.9	Gymnasium	10.4	6.6	3.7
Sundae	8.9	5.1	3.7	Somersault	9.4	5.6	3.7
Largely similar estimated AoAs							
Trespass	9.2	9.2	0	Sibling	7.6	7.6	0
Obsession	9.6	9.6	0	Primate	9.3	9.3	0
Digestible	9.5	9.5	0	Benefit	9.2	9.2	0
Horrid	8.5	8.5	0	Interchange	9.9	9.9	
Descriptive	7.9	7.9	0	Visualize	9.9	9.9	0

There are some words understood at a reasonably young age but acquired in print a few years later. For example, Kuperman et al. (2012) participants rated *mother* and *potty* as quite young, but the current data suggest they might be read only about age 6–7. This is 1–2 years after most children begin formal reading instruction, so it may not be implausible for multi-syllabic words with digraphs such as ‘mother’, despite being understood in oral language earlier. Words that participants rated as having quite different written AoAs are difficult-to-spell words such as *spaghetti*, *gymnasium*, and *somersault*, even though children might understand these words by ages 4–6 according to the Kuperman et al. (2012) norms. A pattern amongst words with similar AoAs (e.g., *horrid*, *primate*, *digestible*, *sibling)* seems to be that they are later-acquired words, conceptually more complex, and more associated with academic literacy. They are likely acquired from print at school.

We expected differences between print estimates and the Kuperman et al. (2012) norms to be greater for words with lower AoAs in Kuperman et al. (2012). This is because: a) the estimates reflect auditory word learning, and b) estimates become more similar to print as children get older and begin to acquire most new words from print. We conducted two analyses on directional differences (reading AoA - Kuperman AoA, and writing AoA - Kuperman AoA). First, we correlated directional difference values with the Kuperman et al. (2012) values using Spearman’s rank correlation. A strong inverse correlation was found for reading (*r* = – 0.667, *p* <.001) and writing (*r* = – 0.646, *p* <.001), indicating that as Kuperman et al. (2012) AoA values increased, the difference with print AoA decreased, with early acquired words having higher print estimates. Second, we compared via a median split of the Kuperman et al. (2012) norms the earlier (below median) and later (above median) AoA print estimates using Mann–Whitney *U* tests. For reading, the mean difference for the lower half was 1.101 and the upper half was – 0.095 (*U* = 25,534,246, *p* <.001); for writing, the mean absolute difference was 1.739 for the lower half and 0.627 for the upper half (*U* = 25,278,817, *p* <.001). These results suggest larger print differences for earlier-acquired words.

#### Comparisons with other norms

This section correlates the new print norms with Kuperman et al. (2012), AoE values (Botarleanu et al., 2022), the Glasgow Norms (Scott et al., 2019), the VxGL (Flor et al., 2024), and Brysbaert and Biemiller’s (2017) test-based estimates. To give each source equal weight, observations are limited to the 3155 words common in each dataset. All correlations are significant at *p* <.001.

**Fig. 3:**
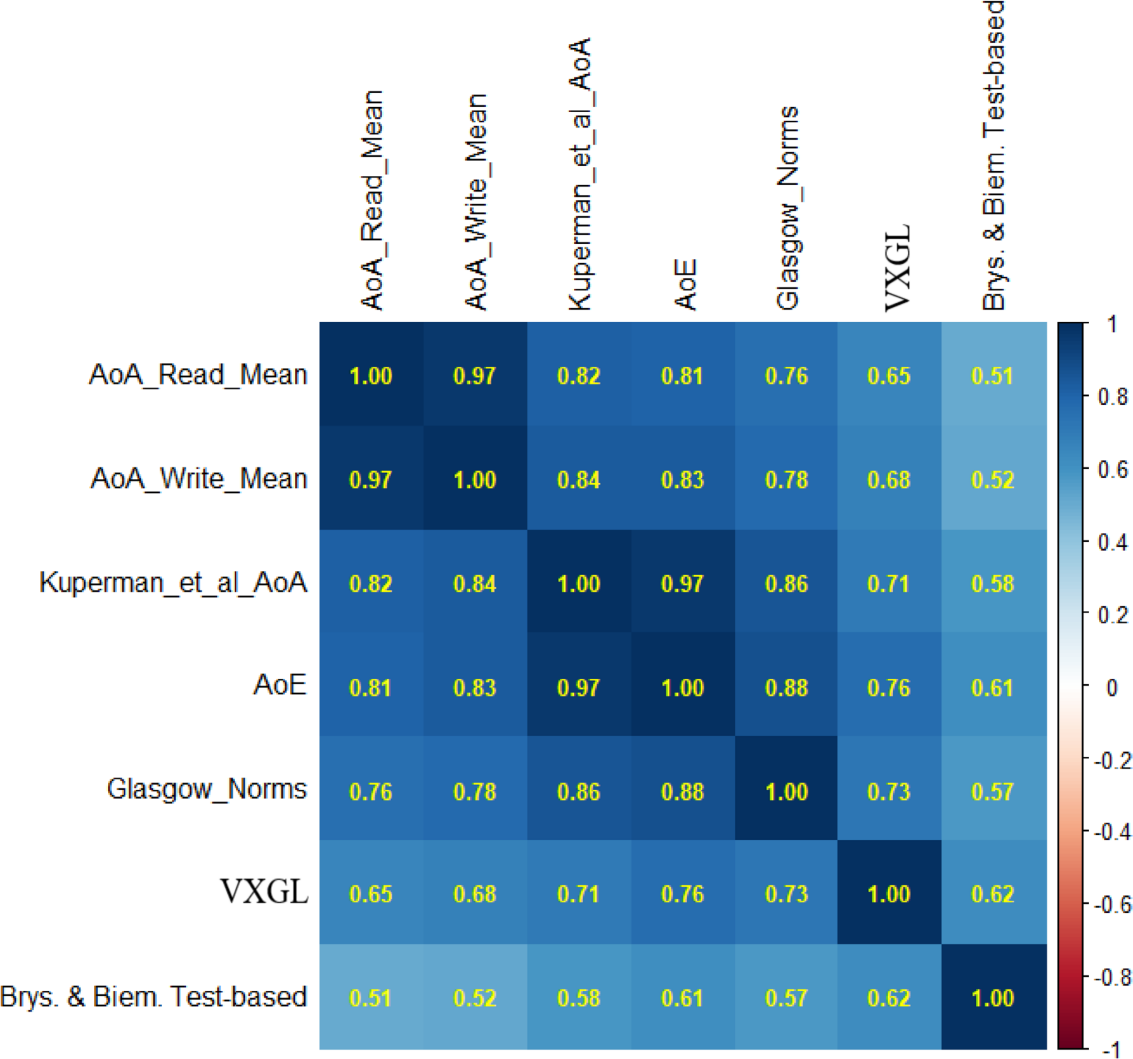
Correlations between AoA estimates (*N *= 3155). *Note. *AoA read mean (this study), AoA write mean (this study), Kuperman et al. AoA (2012), AoE (Botarleanu et al., 2022), Glasgow Norms (Scott et al. 2019), VXGL (Flor et al., 2024), Brysbaert & Biemiller (2017) Test-based

Figure 3 shows that the new crowdsourced norms correlate strongly with the Kuperman et al. (2012) norms (*r* =.82 for reading, *r* =.84 for writing). Since the crowdsourced data was initially checked for correlation of at least.25 with Kuperman et al. (2012), it is important that the print norms also show strong correlations with the other norms, and within the typical range expected when validating new AoAs. In particular, they correlate strongly with AoE (.81 reading;.83 writing). As AoE values derive from print-based NLP, high correlations are both expected and consistent with the validity of the new print estimates. The print norms also correlate well with the Glasgow norms, though less so with VxGL and Brysbaert and Biemiller (2017). The very high correlations between the new reading and writing estimates could indicate that participants have difficulty separating the two, and/or reflect an order effect in the instrument (that is, since participants estimated reading age first, they may have consistently estimated writing AoA as similar but slightly higher).

The Kuperman et al. (2012) and AoE norms correlate strongly with each other, as do the new reading and writing norms. The AoE and Glasgow Norms correlate well with Kuperman and slightly better than the new print norms. One caveat is that Kuperman et al. (2012) estimates were available when the others were collected, and this may have leaked into the analysis (and results) of those studies. For example, the prompts used in the Glasgow norms were similar to the Kuperman et al. (2012) prompt. The older estimates which formed the basis of Brysbaert and Biemiller (2017) have the lowest correlations with all sources and so it is perhaps not recommended to use those estimates anymore.

### Discussion

In Study 1, we conducted a crowdsourcing megastudy that collected and validated new AoA ratings for 11,074 earlier acquired words from Kuperman et al. (2012). We were particularly interested in how estimates from Kuperman et al. (2012) compared to AoAs that specifically asked for reading and writing estimates. As expected, estimated ages for print were higher than AoA estimates in Kuperman et al. (2012). The new crowdsourced data correlated strongly with the original norms, and also exhibited consistently high correlations with other available estimates. The new norms extend the original Kuperman et al. (2012) norms. They are likely to be of value when disambiguation is needed between when words are first understood in oral language and when they can be read or written. In addition, a benefit of these newly crowdsourced norms is that they have not yet been released online so they can function as a test case for the validity of AI-generated estimates, while ruling out contamination. This validation is performed in the next study.

## Study 2: GPT-4 estimates of age of acquisition

Study 2 investigated the extent to which AI-generated AoA estimates could replicate the results of the crowdsourcing in Study 1. By leveraging the query-based nature of LLMs, researchers may be able to extend data from crowdsourcing research to efficiently collect AI-generated AoA data that are a useful proxy of human-generated estimates. Study 2 used a ‘zero-shot learning’ approach, meaning that the AI infers from the conversational prompt without the need to train or fine-tune the LLM.

### Method

Code was written in Python to replicate the instructions used in the crowdsourcing. The code called GPT-4o through the API version [2024-10-21] (https://openai.com/). All code is provided in the OSF (https://tinyurl.com/3ck778nr). The LLM was given the same 11,074 words as input that AMT participants received. We closely replicated Study 1’s instructions in the AI conversational prompt:

“The age of acquisition (AoA) of a vocabulary item refers to the age when a word was first learned. We want you to provide estimates of the print age of acquisition for a target word: Specifically, when a child could first read the word on the page. For example, some words may have been understood by a child when heard in spoken language, but were only learned to be read at a later age. Please estimate the average reading age of acquisition (AoA) of the words listed in ‘{word}’. The estimate can include decimal places. When you are estimating your reading acquisition age for a word, we mean the age at which you think people would have understood that word if they read it in print. Simply print one AOA estimated number next to the word. Output format should be ‘Word: age’. Temperature is set to 0. Estimates should assume a native speaker of English.”

For AI-generated writing, the prompt was altered to elicit “the age at which people are most likely to have learned to spell the words”. We additionally generated estimates aimed at replicating the original Kuperman et al. (2012) norms, thereby providing an additional benchmark for evaluating the AI results. For this, the original Kuperman et al. (2012) request prompt was given to the AI asking “when people would have first understood that word if somebody had used it in front of them, EVEN IF THEY DID NOT use, read, or write it” (Kuperman et al., 2012, p. 980).

The temperature was set to zero, which is an index of how creative the AI should be in its output. Lower values set the AI to be more deterministic in generating the most likely response and allow for better reproducibility. Following Brysbaert et al. (2025) and Martínez et al. (2025a), the code made separate calls to GPT-4o for every word. This reduced prompt dilution and the influence of other words on judgments. In the development stage of the code, to reduce project costs, we initially tried batch processing. We found that while high reliability could be obtained across separate runs, correlations amongst outputs from separate runs would reduce if the word order in the input batch varied. This suggested contextual influence from other words in any given batch. Although processing each word individually increases demand on project resources, it allows for reproducibility.

### Results

#### Descriptive statistics

Table 5 presents the descriptive statistics for the AI-generated estimates for the words from Study 1.

**Table 5:** Descriptive statistics for AI-generated norms

Descriptives	Mean	Std. Dev.	Min.	Max.
AI AoA Kuperman et al.	7.63	2.37	1.50	18.50
AI AoA Reading	9.18	1.95	3.50	16.50
AI AoA Written	9.66	1.74	4.00	18.50

Comparing the Table 5 and Table 3 data, we see the AI-generated estimates are higher on average than the crowdsourced norms: For reading, about 12 months (AI: 9.18, crowdsourced: 8.20), and for writing about 10 months higher (AI: 9.66, crowdsourced: 8.88). Interestingly, the prompt designed to replicate the Kuperman et al. (2012) instructions provides a similar average (AI: 7.63, original: 7.70).

**Fig. 4:**
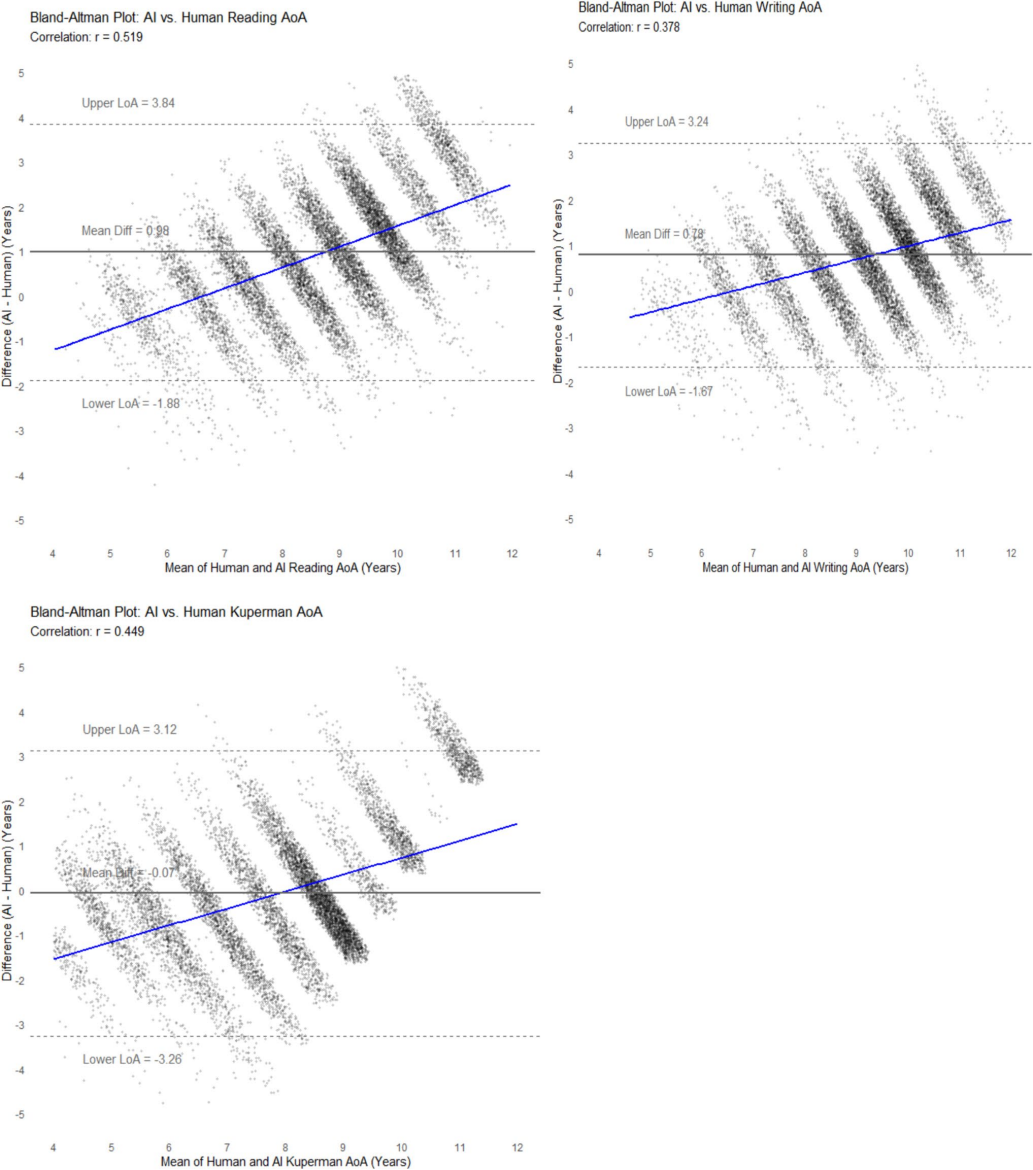
Bland–Altman plots: AI and human estimates

The Bland–Altman plots in Fig. 4 compare AI-estimates for reading, writing, and Kuperman et al. (2012) against human estimates. The horizontal lines indicate the mean difference between AI estimates and human ratings; blue regression lines indicate trends in the differences as a function of the mean AoA.

The upward-sloping patterns and positive mean differences of the Bland–Altman plots indicate the AI tends to produce higher AoAs, particularly for words at the upper end. For reading, estimates are 0.98 years higher on average, with substantial variability (upper LoA 3.84, lower LoA, – 1.88). Written AoA shows a smaller difference (0.78 years) and less variability (LoA range = 4.91 years), indicating better agreement. For the Kuperman et al. (2012) prompt, the AI has negligible bias (– 0.07 years) on average, but the widest LoA range (6.38 years). The visualizations indicate the AI tends to group word-estimates into distinct bands, rather than reflecting a more natural, gradual progression of how children acquire words over time. These patterns may reflect the AI’s limited ability to capture the full variability of human experience (Conde et al., 2025). Rather, the AI estimates of AoA may rely on shared features amongst words in training data (e.g., frequency, text dispersion, morphological complexity, semantic similarities, and connections, etc.). Nonetheless, the Study 2 data overall suggest that while AI estimates systematically differ from human estimates, there is reasonable alignment with human estimates, likely sufficient for a range of applications across the learning sciences.

### Correlations with Study 1 and other norms

**Fig. 5:**
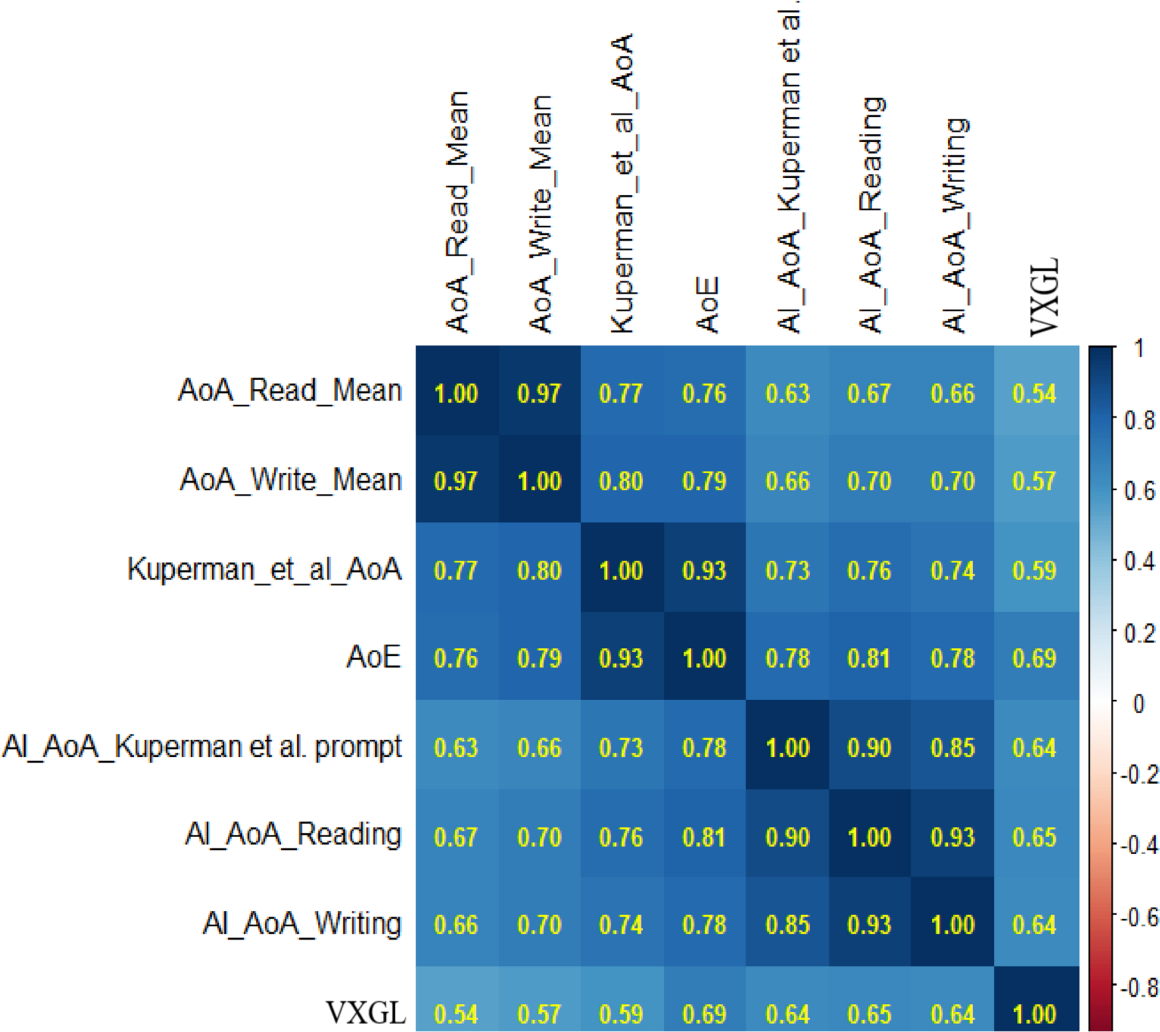
Correlations between AI-generated and other AoA estimates (*N *= 9937). *Note*. AoA read mean (this study), AoA write mean (this study), Kuperman et al. AoA (2012), AI AoA Reading (this study), AI AoA Writing (this study), AI AoA Kueprman et al. prompt (this study), AoE (Botarleanu et al., 2022), VXGL (Flor et al., 2024)

Figure 5 repeats the correlational analysis performed in Study 1 amongst AoA databases with the inclusion of the AI-generated estimates (overlapping words = 9937). To retain a larger sample, the Brysbaert and Biemiller (2017) and Glasgow norms are excluded. All correlations are significant at *p* <. 001.

The AI-generated estimates correlate well with the crowdsourced estimates of Study 1 (reading, *r* =.67, writing,* r* =.70) as do the AI and original Kuperman et al. (2012) estimates based on the same prompt (*r* =.73). The three AI-generated estimates cluster with high intercorrelations, and correlate highly with the AoE (reading, *r* =. 81; writing, *r* =.78). The VXGL (Flor et al., 2024) estimates tend to correlate least with the other measures.

**Fig. 6:**
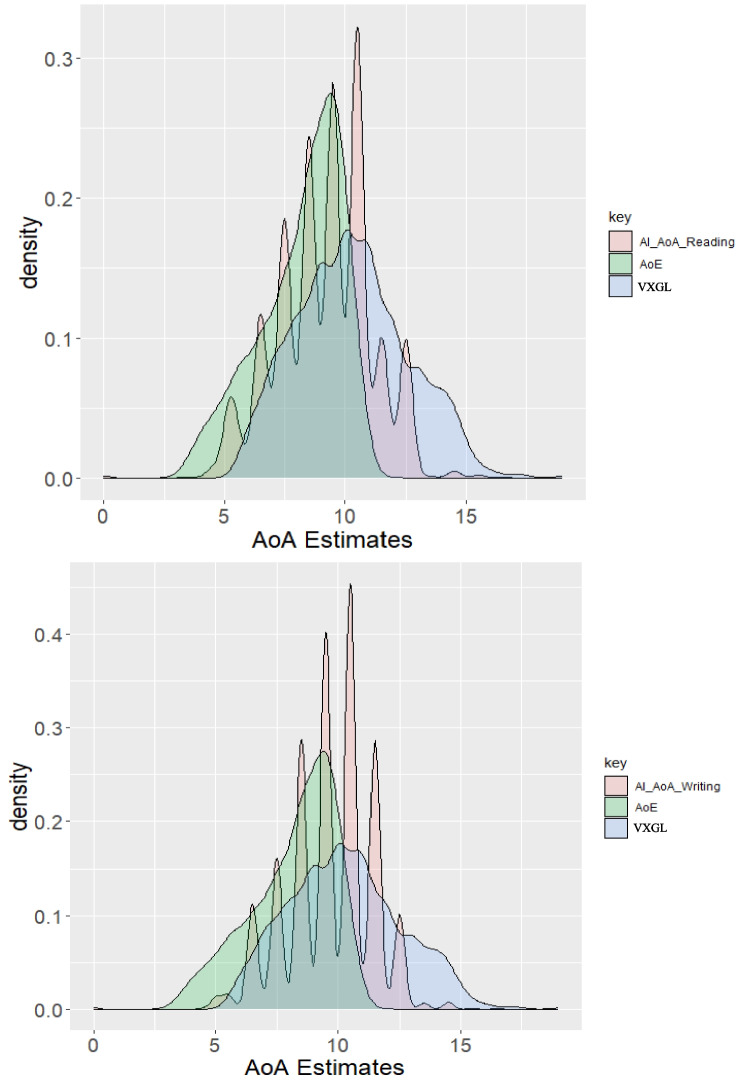
Density plot of AoA distributions: AI reading, AI writing, AoE, and VXGL

It is interesting to plot the distribution of estimated AoAs from the AI and the VXGL and AoE since these norms were not directly generated from human ratings. The panel in Fig. 6 shows these distributions. Given that the VXGL values are estimated grades from 0 (kindergarten) to 16 (4th year of college) (Flor et al., 2024), we converted these to estimated AoAs by adding 5 to each value (i.e., grade 1 having an average age of 6 in the US).

Figure 6 shows that similar to individual human judgments, the AI tends to select whole numbers as age estimates (both the AI and crowdsourcing prompts allowed for estimates between years). This appears to be more pronounced when the AI was estimating writing AoA (lower panel). AI values are distributed over more years than the human ratings in the density plots of Study 1. In particular, the distribution range looks somewhat similar to the VXGL. Since the VXGL maps onto instructional grades in US schools, this may indicate the usefulness of AI estimates for selecting obtaining judgments of grade-level appropriateness for vocabulary.

#### AI-generated estimates and word processing efficiency

In addition to correlations with crowdsourced and other norms, another way to validate AI-generated estimates is to see how well they predict word processing times. We used the values from the English Crowdsourcing Project/ECP (Brysbaert et al., 2019; Mandera et al., 2020) because it is the largest crowdsourced megastudy with the greatest diversity of participants. It consists of responses from 700,000 people about which words they know (word prevalence) in over 62,000 words.

Table 6 shows the percentages of variance accounted for in accuracy and lexical decision time (LDT) from the ECP. The baseline model is frequency from SUBTLEX-US (Brysbaert & New, 2009) and word length. The latter two variables are selected as they are amongst the ‘big five’ and often thought to have precedence over AoA in predicting word processing efficiency. The table first reports the baseline model, then each row is a separate model investigating the change from the AoA norms in Study 1, Study 2, Kuperman et al. (2012), the AoE and VxGL.

**Table 6:** Percentage of variance accounted for in ECP word accuracy and speed by AoA norms (*N* = 9920)

	Reaction time				Accuracy			
	*R*	*R*^2^	*R*^2^ Change	Sig.	*R*	*R*^2^	*R*^2^ Change	Sig.
Freq. + Length	.647	.418		.001	.452	. 204		.001
+ AoA Kuperman et al. (2012)	.648	.419	.001	.001	.452	. 204	0	.068
+ AoA Reading (Study 1)	.649	.422	.004	.001	.452	. 204	0	.019
+ AoA Writing (Study 1)	.649	.421	.003	.001	.452	. 204	0	.055
+ AI Reading AoA	.652	.425	.007	.001	.459	.210	.006	.001
+AI Writing AoA	.653	.426	.008	.001	.459	.211	.007	.001
+ AI Kuperman et al. (2012)	.653	.426	.008	.001	.459	.211	.007	.001
+ VXGL	**.662**	**.438**	**.020**	**.001**	**.468**	**.219**	**.015**	**.001**
+ AoE	.651	.424	.006	.001	.455	.207	.003	.001

Table 6 shows that the baseline model explained 41.8% of the variance in reaction time (*R*^2^ =.418, *p* <.001) and 20.4% of the variance in accuracy (*R*^2^ =.204, *p* <.001). The addition of various AoA measures improved the model’s explanatory power with small additional effects. The Study 1 norms perform slightly better in accounting for visual word processing than the original Kuperman et al. (2012) norms for early acquired words. The AI estimates perform slightly better than human ratings. The VXGL estimates perform best overall in the regressions. Noteworthy is that the percentages of variance accounted for are low for accuracy, with the crowdsourced norms making no significant contribution. This could be understood from the fact that the analysis is limited to approximately 10,000 relatively easy words.

#### AI-generated estimates and readability

To explore predictive validity, we computed correlations between the average AoA of content words in texts within the CommonLit Ease of Readability (CLEAR) corpus (Crossley et al., 2023). This corpus contains readability scores for over 5000 text excerpts from grades 3–12. We hypothesized that the average AoA of words in these texts should increase by grade and reading difficulty.

Code was written to compute the average AoA of all content words in each text of CLEAR. The code used the Perceptron Tagger (NLTK, Python) and provided the mean AoA of all nouns, verbs, adjectives and adverbs (tagset: ‘NN’, ‘NNS', 'NNP', 'NNPS', 'VB', 'VBD', 'VBG', 'VBN', 'VBP', 'VBZ', 'JJ', 'JJR', 'JJS', 'RB', 'RBR', 'RBS'). We correlated this average AoA value with the following readability measures: Bradley-Terry (BT-easiness) score, a gold-standard readability metric; the Flesch-Kincaid Grade Levels and Lexile Bands; and two NLP measures, the CAREC (Crowdsourced Algorithm of Reading Comprehension) and the CML2RI (Coh-Metrix L2 Readability Index) (Crossley et al., 2008; Graesser et al., 2004). Lower-grade texts have lower Lexile and Flesch–Kincaid scores. Easier-to-read texts have smaller CAREC values. Conversely, BT-easiness and CML2RI assign higher scores to easier-to-read texts. Pearson correlations are reported in Table 7. All correlations are significant at *p* <.01.

**Table 7:** Correlations between readability (K-12) and mean AoA of words in CLEAR texts (*N* = 4692)

	Lexile band	Flesch–Kincaid G-L	CAREC	BT easiness	CML2RI
AoA reading	.582	.563	.710	–.522	–.607
AoA writing	.595	.574	.730	–.532	–.613
AoA Kuperman et al.	.**612**	.588	.756	**–.558**	–.613
AI reading	.600	.592	.746	–.542	–.593
AI writing	.598	.598	.748	–.531	–.581
AI Kuperman et al.	.593	.580	.756	–.529	–.599
VxGL	.586	.577	**.770**	–.543	–.600
AoE	**.612**	**.593**	.759	–.557	**–.620**

The average AoA for content words in CLEAR texts showed moderate-to-strong correlations with readability and grade-level appropriateness. Higher-grade texts, indicated by the Lexile and Flesch–Kincaid measures, contained words with higher AoAs. Similarly, texts considered harder to read had higher AoA values judged by CAREC, Bradley–Terry easiness, and CML2RI readability measures. Correlations between AoE and readability tended to be stronger. Overall, the data confirm expectations that the average AoAs for both human and AI-generated AoAs are higher in texts assigned grades and reading difficulty in the CLEAR corpus.

### Discussion

Study 2 investigated AI-generated AoA estimates for the words in Study 1. These estimates correlated.65 to.75 with the crowdsourced norms of Study 1 and Kuperman et al. (2012). The strength of the relationships indicates that AI-generated estimates provide a useable alternative to crowdsourcing megastudies. The estimates gave slightly higher AoAs on average than human estimates for print, though less so when given a prompt similar to Kuperman et al. (2012). This might be because the Kuperman et al. (2012) dataset was available online when GPT-4 was trained, whereas Study 1 was not available to inform the LLM.

AI-generated estimates were useful predictors of word recognition performance. They were on par with human ratings in accounting for processing speed and accuracy. Values from the VXGL performed better. The Study 2 and Study 1 AoAs also robustly correlated with text readability and grade level expectations, although we here see that the human ratings were better predictors than the AI estimates.

The overall findings of Study 2 confirm that the GPT4-based method is a way to obtain AoA measures comparable to crowdsourced methods, and that good approximations of human estimates can be obtained through zero-shot learning approaches using conversational prompts similar to those used with human participants.

## Study 3: Extending the AI-generated AoA norms to new stimuli

Study 3 built on the method of Study 2 to extend available AoA norms with AI. Firstly, zero-shot estimates for all familiar words in the ECP were obtained. The goal was to generate AI estimates for the substantial number of ECP words that do not have AoA estimates in Kuperman et al. (2012). As noted in Study 2, the ECP contains 62,000 words. While it is tempting to release AoAs for all these words, Sendín et al. (2025) convincingly argued that AoA information only makes sense for words familiar to most people, as AoA represents the acquisition order of *known* words. For words that are not generally known, word familiarity is a better index. Therefore, we only release estimates for the set of 25,079 words known by 95% or more of the participants in the ECP.

Secondly, Study 3 investigated the potential for model finetuning to improve the alignment between AI-generated and human ratings. Sendín et al. (2025) found estimates in Spanish converged more after fine-tuning on 2000 examples (with little further improvement expected from additional training data). We took 28,054 words from the original Kuperman et al. (2012) norms (words with GPT-4 familiarity estimates greater than 3 (Brysbaert et al., 2025)) and trained a model on 2000 of these estimates. The fine-tuned model was then deployed to derive AoA estimates for all familiar words in original Kuperman et al. (2012) database and the ECP. This also allowed us to compare the zero-shot and fine-tuned ratings and evaluate whether any improvements in alignment in English were similar in scale to those found for Spanish.

In Study 1, we deliberately limited crowdsourcing of print norms to an age range corresponding to elementary school. However, there is potential value in having print estimates for words acquired across the secondary school years. The resources/costs of a large-scale crowdsourcing study, however, may outweigh the benefits, so AI is a good solution here. Study 3 therefore additionally derived print estimates for all familiar words in Kuperman et al. (2012) and the ECP.

### Method

For the zero-shot approach, the same code, process and prompts as Study 2 were used. Each familiar word in the ECP word was given to the AI via separate API calls. Monitoring of the output indicated some errors could occur. Comparisons of output to word input found cases of ‘hallucination’ (e.g., the input word 'restrictive' was printed as 'restriptive' and given an AoA estimate in the output file). These were corrected by re-entering the words into the AI. A handful of taboo words were consistently refused by the AI. It was felt that it was unnecessary to obtain AoAs for these words and so they have missing values in the database provided in the OSF.

For the fine-tuned approach, a random sample of 2000 words was taken from Kuperman et al. (2012). The sample covered the full range of age values in the database. The json file used for training is available in the OSF. The AI was provided with the Kuperman et al. (2012) prompt, a training word, generated its AoA estimate, then given the actual estimate as feedback (e.g., "Word: comprehensible, AoA: 11.22"). The model updated connectionist weights across 1500 iterations.

In the zero-shot approach, we processed the same wordlist three times through the AI to generate estimates for reading, writing, and the Kuperman et al. (2012) prompt. By the time we were in the process of deploying the fine-tuned model, we had established consistently high correlations amongst AI runs when separate prompts for Kuperman et al. (2012), reading and writing were used. To use remaining research funds appropriately, we adapted the prompt to obtain three AoA estimates at once, beginning with the Kuperman et al. (2012) prompt:

"The age of acquisition (AoA) of a vocabulary item refers to the age when a word was first learned. We want you to provide estimates of the AoA for a target word. Firstly, estimate when people would have first understood the word if somebody had used it in front of them, EVEN IF THEY DID NOT use, read, or write it at the time. Secondly, estimate the AoA for when people could first read the word on the page. For example, some words may have been understood by a child when heard in spoken language, but were only learned to be read at a later age. Thirdly, estimate the AoA for when a child could first write a word with accurate spelling. For example, some words may have been understood by a child when heard in spoken language, or read on a page, but were only learned to be spelled correctly at a later age. Estimates can include decimal places. Estimates should assume a native speaker of English. Temperature is set to 0. Now, estimate the AoA of {word}'. Output format: 'Word: {word}, Kup AoA: <number> READ AoA: <number> WRITE AoA: <number>'"

Each word was still given to the AI via separate API calls, but similar to Study 1 it should be acknowledged that this prompt has potential order effects (e.g., the first rating influencing the second and third). Also, less than ideal is that the fine-tuned model was GPT-4.1., while zero-shot estimates came from GPT-4o as in Study 2. The university where processing was done updated subscriptions during Study 3 and retired GPT-4o. This occurred after the zero-shot data had been collected. It did not seem environmentally friendly to regenerate all zero-shot estimates with 4.1, as they were expected to correlate.99 with those of 4 anyway and good outcomes had been demonstrated from the earlier model. The goal was to explore how model finetuning might improve alignment with human norms, not compare models. For these reasons, we also did not collect zero-shot estimates for the subset of words in Kuperman et al. (2012) that are not attested in the ECP as we could not see that this would add much value to Study 3 or the suite of resources we aimed to share. Still, it is good to keep in mind that observed differences between untrained/trained LLM estimates may be slightly due to improved performance of 4.1.

## Results

### Descriptive statistics and estimates for new words

Table 8 reports descriptive statistics. To better understand the differences between zero-approaches and finetuning, central tendencies are reported for all shared words in the ECP (Mandera et al., 2020) and Kuperman et al. (2012) (*N* = 18,796). To evaluate how closely the fine-tuned estimates approximate the Kuperman et al. (2012) norms, descriptive statistics are reported for this larger set (*N* = 28,054).

**Table 8:** Means, standard deviations for AI estimates (zero-shot and fine-tuned)

Shared words in ECP and Kuperman et al. datasets (*N* = 18796)				
	Mean	Std. Dev.	Min	Max
AI Kuperman et al. 2012 AoA Zero-shot	9.67	3.28	1.50	25.5
AI Kuperman et al. 2012 AoA Fine-tuned	9.35	2.25	2.00	15.0
(Actual) Kuperman et al. (2012)	9.74	2.69	1.89	18.14
AI Reading AoA Zero-shot	10.81	2.48	3.50	24.5
AI Reading AoA Fine-tuned	9.70	2.31	3.00	15.5
AI Writing AoA Zero-shot	11.14	2.30	4.00	24.5
AI Writing AoA Fine-tuned	10.08	2.38	3.00	15.8
Fined-tuned versus actual Kuperman et al. dataset (*N* = 28054)				
(Actual) Kuperman et al. (2012)	10.67	2.94	1.58	21.00
AI Kuperman et al., 2012 AoA Fine-tuned	10.14	2.47	2.00	16.00
AI Reading AoA Fine-tuned	10.52	2.52	3.00	16.50
AI Writing AoA Fine-tuned	10.91	2.57	3.00	16.80

Table 8 shows that as with Study 2, GPT estimates writing AoAs as highest, followed by reading and Kuperman et al. (2012) prompt estimates the lowest. Comparing the zero-shot and fine-tuned models across the ECP words, both models have an overall average that is similar to Kuperman et al. (2012). For print, the fine-tuned estimates are lower than the zero-shot estimates. However, the range of values in the trained model was reduced, perhaps because of fitting to the training data which has fewer examples of higher AoA vocabulary. If we consider only average AoA for words under age 10 in the fine-tuned Kuperman et al. (2012) data we find: (Reading) Study 3 = 8.11 cf. Study 1 = 8.20 cf. Study 2 = 9.18; (Writing) = 8.44 cf. Study 1 = 8.88 cf. Study 2 = 9.66). So, for early-acquired words, the finetuning model provided print estimates closer to Study 1.

#### Correlations with previous norms: Zero-shot and fine-tuned models

**Fig. 7:**
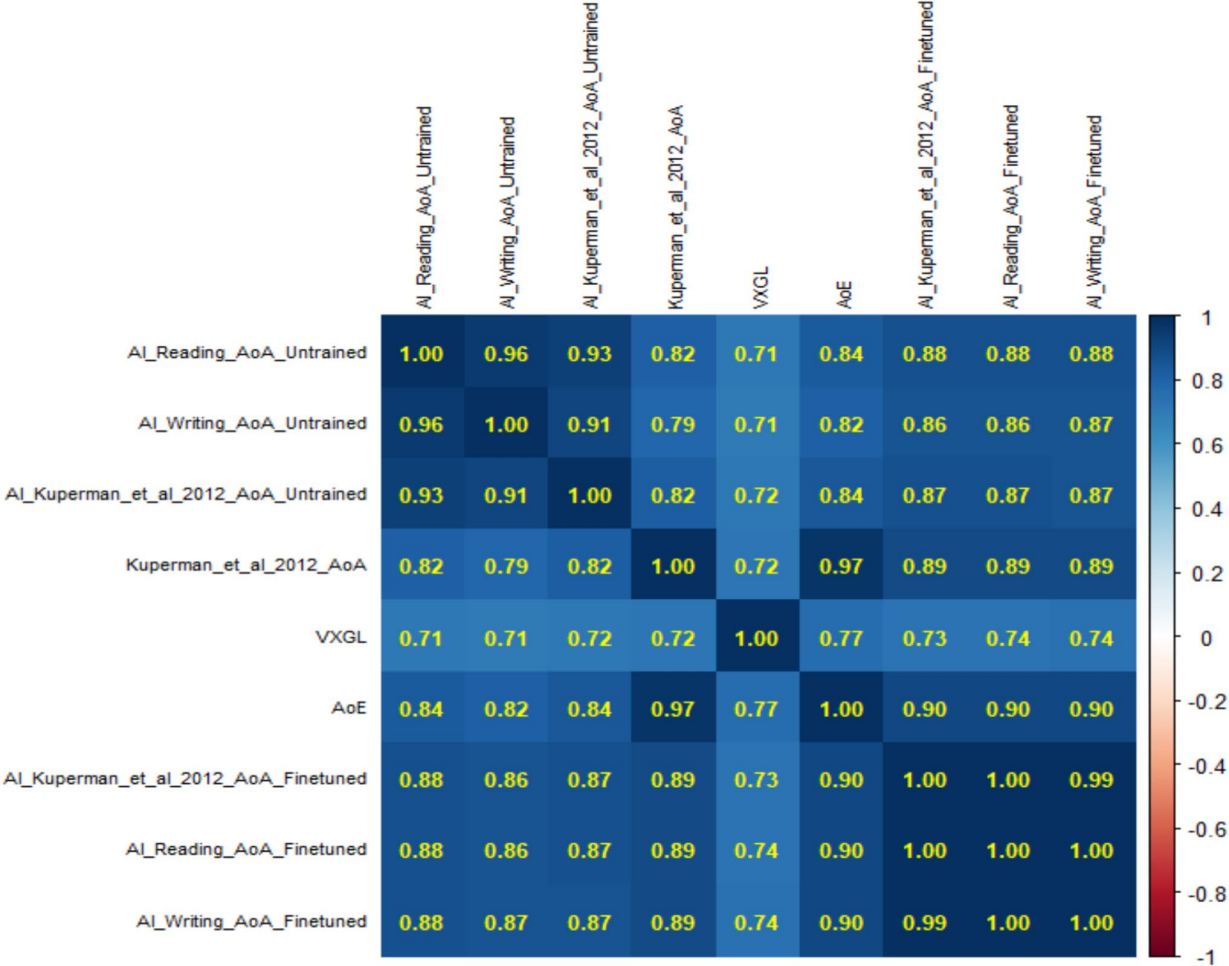
Correlations amongst AI, ECP, Kuperman et al. (2012), VXGL, AoE (*N* = 17,680)

Study 3 provides a larger number of AI-generated estimates than Study 2, allowing for a more robust investigation of relationships with word processing and other large AoA databases. Figure 7 shows the correlations between the untrained AI, Kuperman et al. (2012), VXGL, AoE, and the fine-tuned model. As expected, because of the larger range, the correlations increased to around.85 (instead of.75). All correlations are significant at the *p* <.001 level.

Figure 7 shows that for words shared across the datasets, both the zero-shot and fine-tuned models correlated well with human estimates, the VxGL and AoE. Fine-tuning led to improvements across all correlations. For example, the zero-shot estimates correlated *r* =.82 with Kuperman et al. (2012) versus *r* =.89 for the fine-tuned model. The correlation for all 28,054 fine-tuned estimates in the OSF and their actual Kuperman et al. (2012) ratings is *r* =.92. This is close to what can maximally be expected given that the reliability of the Kuperman et al. (2012) ratings is around.9.

Correlations of the independently derived zero-shot AoA estimates in Study 3 and Study 2 for overlapping words were *r* =.98 for reading and writing and *r* =.99 for the Kuperman et al. (2012) prompt, indicating high reproducibility. They also confirm that estimates are not affected by the sequence of words given to the AI when the prompt is repeated before each word.

Perhaps the most interesting finding is that because the untrained model already correlates very well with the human ratings, limited progress was made with finetuning a model. For example, correlations in Fig. 7 show an increase from.82 (zero-shot) to.89 (fine-tuned), and this includes potential gains from model upgrades. Similar modest correlational increases are observed even when analyses were restricted to words shared across Studies 1, 2, and the finetuning of Study 3 (*N* = 10,925). For example, the crowdsourced reading estimates increase was from *r* =.69 (zero-shot) to *r* =.71 (fine-tuned) and writing from *r* =.72 (zero-shot) to *r* =.75 (fine-tuned). This suggests that finetuning is less needed for English AoAs than in Sendín et al.’s (2025) study of Spanish. Having said that, a change from.82 to.89 is a 12% increase in *r*^2^ from.67 to.79, which is not insubstantial, so fine-tuning in English remains valuable in improving alignment with human-derived psycholinguistic norms (Conde et al., 2025).

#### AoA estimates for new words

The main appeal of AI-generated estimates is that they can easily be obtained for words without existing human norms. Not all words in the ECP have AoAs available in Kuperman et al. (2012). From GPT-4o we have obtained AI estimates for 6183 new familiar words. Table 9 provides examples of words in particular age ranges now added to Kuperman et al. (2012).

**Table 9:** New words in age ranges added by the AI for the ECP (zero-shot)

AoA	*N* in Kuperman et al. (2012)	*N* added AI	Examples: AI-generated additional words
≤ 2	9	3	Crawling*…
3	122	11	Hi, an, eating, poo, are…
4	468	84	Bouncing, went, feet, teddy, wearing…
5	846	242	Cuddly, broken, lost, slid, caught, worse…
6	1203	362	Hung, melting, probably, nope …
7	1640	436	Bendable, woody, honestly, nosey …
8	2237	337	Appealing, dimly, grubs, initials, lice…
9	2941	1331	Aqua, submerged, farthest, peckish, stranded…
10	3406	219	Fleeting, larvae, polluted, sparing, strewn …
11	3850	634	Algae, blustering, cosmetics, faltering, italics, media…
12	3837	22	Degrading, irrationally, obnoxiously, rashly …
13	3600	1324	Bedazzle, cordially, elapse, levy, thereafter, vexed…
14	2854	2	Cyberbullying, Facebook…
15	1988	676	Verbatim, transfigure, annotation, conversely …
16	1057	137	Zen, woke, accredit, biofuel, extenuating …
17	511	313	Reprehensibly, tweaker, stakeholder, uninsurable
18	166	1	Graduating
19	44	44	Macroeconomics, fresher, synergize, electrocardiography …
20+	19	5	Functionalist, microstructural, neurophysiological…

* The fine-tuned estimate for ‘crawling’ is age 4

Table 9 shows that for some age bands, the AI did not add as many words as it did in other age bands. The reasons are unclear to the authors, but, as shown, fewer words have been added, for example, to ages 12 and 14 compared to 13 and 15. It is also worth considering, as we did in Study 2, the patterns of divergence and potential error with regard to human ratings. Table 10, for example, is similar to Table 4 but with examples of words where the AI gave similar AoAs to Kuperman et al. (2012) and words with wide divergence even from the fine-tuned model.

**Table 10:** Examples of words (fine-tuned model) with different and similar AoAs cf. Kuperman et al. (2012)

Substantially different AoA estimates			
	AI AoA estimate (fine-tuned)	Crowdsourced AoA in Kuperman et al. (2012)	Age diff.
Colicky	10	15	5
Sagacity	14	19	5
Snarky	11	16.56	5.56
Amylase	14	18.2	4.2
Twitter	12	18.14	6.14
App	10	18.33	8.33
Similar AoA estimates			
Ambassador	11	11	0
Cadaver	13	13	0
Cotton	6	6	0
Toothbrush	4	4.33	0.33
Incorrect	7	6.65	0.35
Hypotenuse	12	12.36	0.36

Table 10 shows alignment for many words across different age spans (e.g., ‘*incorrect’*, ‘*toothbrush’*., *hypotenuse’)* but also differences of up to 8 years in the case of ‘*app’*. This data is interesting. One suspects potential AI ‘errors’ relate to its training. For example, ‘colicky’ is rated by the AI as being acquired 5 years before its Kuperman et al. (2012) estimate, at the age of 10. This AoA is, at face value, not overly convincing. On the other hand, one suspects a possible explanation for other mismatches such as ‘Twitter’ and ‘app’ may not be an error but reflect a change in language use over time since Kuperman et al. (2012). Over the past 10–15 years, Internet technology and language have been embedded in the lives of younger children, and the participants in Kuperman et al. (2012) may indeed have learned these words at later ages than the current generation (i.e., Twitter was released about 6 years before the original AoA norms). For other examples in Table 10, such as ‘snarky’, ‘sagacity’, ‘amylase’, it is hard to say why these are approximately 5 years lower than the Kuperman et al. (2012) norms.

**Fig. 8:**
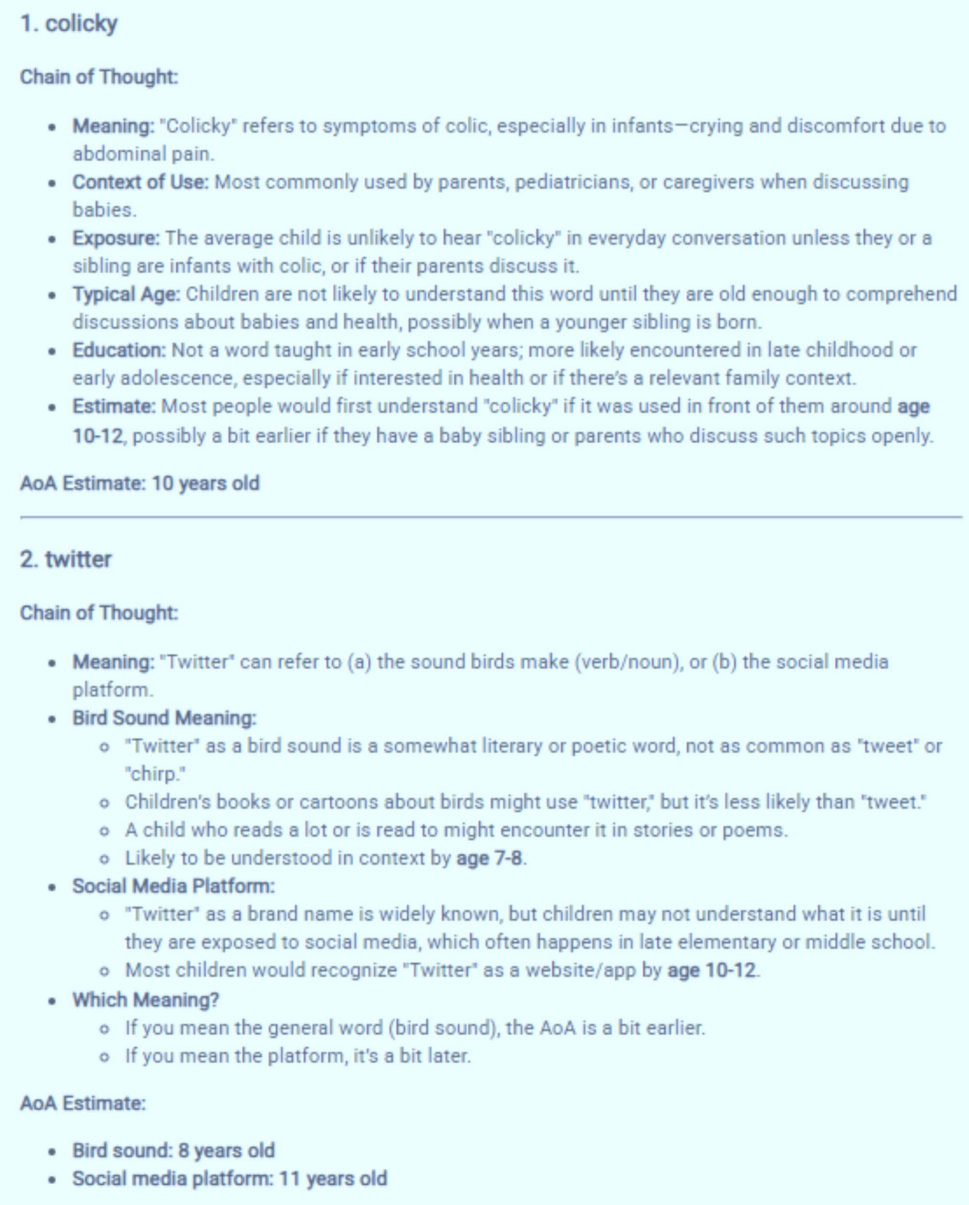
Illustrative CoT (GPT 4.1) when asked to make AoA estimates

Drawing on a suggestion from a helpful reviewer, we thought asking for a Chain of Thought (CoT) might be illustrative. Chain of Thought (CoT) is a prompting technique where users instruct LLMs to detail reasoning. We did not use CoT in the current study, though it is a worthwhile avenue for future research. CoT has been shown to improve performance on certain tasks, but it also has challenges such as increased energy consumption, financial costs, and the problem of ‘faithfulness’, meaning that generated CoT may often not represent the model's genuine decision-making processes (Chen et al., 2025; Arcuschin et al., 2025). Nonetheless, acknowledging these caveats, we thought it interesting to provide GPT-4 with the Kuperman et al. (2012) instructions and ask for a CoT for ‘colicky’ and ‘Twitter’ given that these diverged so much from human data. Figure 8 is the output; we simply used the web interface for this and did not provide it with our estimated ages.

The CoT offered by GPT-4.1 is that ‘colicky’ might come up in family discussions about a younger sibling, and so be understood around age 10–12. However, the AI also reasons that if there is no younger child in the household, the word would likely be acquired much later. For ‘Twitter’, the AI interestingly distinguishes polysemy between the bird sound and the social media platform. The social media app is rated as being acquired about age 10–12, with the CoT mentioning social media exposure in upper elementary school years. The bird sound is suggested to be acquired earlier from children’s books or cartoons. Again, the relationship between AoA predictions from the LLM and such ‘reasoning’ is unknown. The LLM does not have a cognitive model built in. This CoT example, however, does raise the important issue of polysemy. Polysemy is something the current study has not investigated thoroughly, and it is important for future research. The AI could be asked to provide AoA estimates for different senses of a word- a challenging but worthwhile project, as many words have variable senses.

## Relationships with word processing

We considered the implications of model finetuning for the prediction of word recognition. Table 11 repeats the regressions of Study 2 on the larger pool of ECP words now available. The table first reports the baseline model that includes only frequency and length, then the change in the model from additions of the various AoA norms. The dependent variables were RT and accuracy from the ECP.

**Table 11:** Percentage of variance accounted for in the accuracy and speed data of the ECP (*N* = 17680)

	Reaction time			Accuracy		
	*R*	*R*^2^	*R*^2^ change	*R*	*R*^2^	*R*^2^ change
Freq. + Length	0.731	0.534		0.536	0.288	
+Kuperman_et_al_^2012^_AoA	**0.742**	**0.55**	**0.016**	0.575	0.331	0.043
+AI Reading AoA Untrained	0.741	0.549	0.014	0.570	0.324	0.037
+AI Writing AoA Untrained	0.741	0.549	0.014	0.569	0.324	0.036
+AI Kuperman et al. (2012) Untrained	**0.742**	**0.550**	**0.016**	0.572	0.327	0.039
+VXGL	0.739	0.547	0.012	0.566	0.320	0.032
+AoE	**0.742**	**0.550**	**0.016**	0.574	0.330	0.042
+AI Reading AoA Fine-tuned	0.740	0.547	0.013	0.579	0.335	0.047
+AI Writing AoA Fine-tuned	0.740	0.547	0.013	**0.580**	**0.336**	**0.048**
+AI Kuperman et al. (2012) Fine-tuned	0.739	0.546	0.011	0.575	0.331	0.043

Regression outcomes of Study 3 show differences from those of Study 2. There is more variance explained in both RTs and accuracy. The baseline model with frequency and length as predictors explained 53% of the variance in reaction time (*R*^2^ =.534, *p* <.001) and 29% of the variance in accuracy (*R*^2^ =.288, *p* <.001). All AoA estimates added significantly more explanatory power to the baseline model (*p* <.001). While the VXGL performed best in Study 2 for both accuracy and processing time, now all values are very close to each other, including untrained and trained models.

### Discussion

Study 3 generated AI AoA estimates for all familiar words in the ECP and Kuperman et al. (2012) databases. This provided a larger pool of words to test the validity of AI-generated AoA estimates. We additionally explored the effect of model finetuning on 2000 human word ratings. The study confirmed that AI-generated AoA estimates remain valid for later-acquired words. The correlations between the AI estimates and the human ratings were almost as high as the reliability of the human ratings, even for zero-shot prompting without model finetuning. Finetuning on 2000 words improved alignment but overall, it seems finetuning is less needed for English AoA estimates than Sendín et al. (2025) observed for Spanish. This might be understood from the fact that GPT-4 was trained predominantly on the English language. As a result, the performance of the default model is likely to be better for English than for other languages.

Study 3 suggests less of a need for researchers in the field of English language to learn how to train models, which increases access by lowering technical expertise and financial costs. There are also other concerns about finetuning, including that the outcome of the model is only as good as the original ratings (Sendín et al., 2025). Any bias in human ratings is mimicked by the fine-tuned model. As a result, a fine-tuned model can provide less new information than a zero-shot model..

## General discussion

Three related studies extended the AoA norms of Kuperman et al. (2012). Study 1 was a crowdsourcing megastudy that collected new AoA norms for print, contributing 790,024 new ratings for 11,074 early-acquired words. We developed these new crowdsourced norms to supplement the Kuperman et al. (2012) estimates because of the lack of disambiguation in the original norms between oral language and print AoA, an issue of interest for words acquired at an early age in the elementary school years. Now, researchers have access to estimates of when people think they were able to read and write these words. Overall, the order of acquisition was largely the same. The estimated ages of acquisition were higher for printed words, and there were also large differences for some words that are known early in spoken but not in written form.

The new AoA estimates should find a range of uses in research. Consider projects such as Graves et al. (2019), who aimed to identify words that could present reading comprehension challenges for elementary school children. These were identified by extracting higher frequency words in texts prescribed for each grade and then categorizing them into ‘challenging’ wordlists for each grade depending on whether they had AoAs 1-year or above in Kuperman et al. (2012). Since the print AoA are on average higher than the original estimates across the elementary school years (approximately up to age 10), calibrating against the Kuperman et al. (2012) AoAs may underestimate the learning challenge of these words.

The reading and writing estimates of Study 1 are highly correlated (*r* =.97), but there is value in releasing both. As noted, the means differed systematically, with writing estimates higher on average than reading estimates. This pattern is consistent with real-world expectations of meaning-recognition (reading) preceding production (writing) (Biemiller, 2010). Also, given the *r*^2^ =.94, about 6% of the variance is not shared, so modality-specific information is retained. Providing both reading and writing crowdsourced norms maximizes flexibility: researchers can choose the modality most relevant to their use case (e.g., reading for comprehension studies, writing for composition tasks) or combine them as needed, without assuming full equivalence. With thousands of items, 6% unshared variance yields meaningful item-level differences for these early-acquired words.

Study 2 investigated the extent to which AI could replicate the crowdsourced data from Study 1. Results confirmed that AI can provide estimates that correlate strongly with human judgements, which adds to an increasing body of research pointing to the utility of LLMs for developing resources in the behavioral sciences (Brysbaert et al., 2025; Martínez et al., 2025a; Trott, 2024). We have been able to rule out contamination because the new norms of Study 1 had no previous online presence. At the same time, we saw that the estimated ages were considerably higher than the ones using the Kuperman et al. (2012) prompt, where the estimates agreed particularly well with published findings (which could have been used as part of the training materials). Since no training data from the new crowdsourced norms was given to the AI when asked to generate its estimates, this means that in the absence of calibration data, the AI can generate good estimates of AoA.

However, important advantages remain for crowdsourced ratings over the AI estimates. While the AI captured the overall order of word acquisition well (Brysbaert, 2017; Elsherif et al., 2023), ratings can significantly vary at the individual word level from human judgments. This was indicated in particular by the Bland–Altman analysis in Study 2. Some individual word estimates may diverge several years from human-collected data. Given these findings, AI estimates should be carefully and checked against human data when available. The estimates we provide in the supplementary materials, for example, are not suitable for high-stakes applications such as educational testing or clinical contexts. To give an example, appropriate use in an educational context might be teachers’ using AI estimates as information that helps guide their selection of words for vocabulary instruction; inappropriate use would be using AI estimates to test a child’s knowledge of age-appropriate vocabulary and consequently making intervention decisions based on such data.

Study 3 extended the available AoA norms for English to a larger dataset of 25,079 familiar words from the ECP (Brysbaert et al., 2019; Mandera et al., 2020) and added reading and writing estimates to 28,054 words of Kuperman et al. (2012). We first investigated a zero-shot approach, which provided strong correlations with other large datasets of AoA norms similar to those found when validating new human-generated AoA norms against precursors. This is an important finding as it shows that good estimates for the English language can be obtained relatively simply through AI. Simply by querying the AI through a conversational prompt, reliable results can reasonably be obtained.

Finetuning models improved alignment with human ratings, but improvements were found to be less in scale than in the recent study of Spanish by Sendín et al. (2025). This suggests that model training for tasks such as AoA estimation may be less necessary for English. The finding is consistent with the good estimates for familiarity, concreteness, valence, and arousal previously obtained with untrained models in English (Brysbaert et al., 2025; Martínez et al., 2025a; Trott 2024).

Nevertheless, it is useful for researchers to know that better estimates can be obtained after some fine-tuning. The finetuning of Study 3 increased *r*^2^ from.67 to.79. When researchers encounter a variable for which the model does not provide good output, finetuning is particularly important. Such a case is reported in Martínez et al. (2025b), where the authors found that GPT-4o-mini does not provide useful estimates of word lexical decision times with zero-shot prompts, but after fine-tuning on 3000 representative words, it produces results that closely match human performance.

This paper is part of a shift from the era of crowdsourced megastudies to an era characterized by AI-generated norms (e.g., Brysbaert et al., 2025; Martínez et al., 2025a; Trott, 2024). Just as it was initially unclear how well crowdsourced megastudies would be able to provide valuable alternatives to lab-based research data (Kuperman et al., 2012), at present, we are comparing the output of LLMs to that collected in crowdsourcing. The current study adds to the growing consensus that leveraging AI to produce word-related norms is valid and produces an interesting addition to human-collected data.

The availability of AI measures is of particular interest for variables that do not have large-scale human ratings. For instance, Smith et al. (2025) raised doubts about the value of arousal ratings for words because of the low correlation between different operationalizations. Rather than having to run a series of human ratings studies, researchers can first test the output from different prompts generated using LLMs. Even for variables with large-scale human ratings, AI provides a quick estimate for new words and words not available in the existing ratings. Despite the extensive resources provided by Kuperman et al. (2012), we were able to extend the norms with over 6000 words from the ECP. Indeed, the ECP itself is likely to have missed some words. The advantage of AI-generated estimates, particularly with zero-shot prompting, is that they are easy to obtain. If extra AoA data is needed, it is easy for researchers and teachers to use an online interactive interface for an LLM. They do not require the coding skills needed for Studies 2 and 3. We recommend first selecting some of the words made available in the present publication to see if the prompt and the LLM work as we have reported, and then ask for estimates for the new stimuli.

The availability of AI estimates is also of interest for researchers working in languages other than English. For many languages, large-scale human ratings are missing. AI-generated estimates give interesting initial information and future work can investigate which variables are able to be brought to a good level by finetuning the AI model on a representative sample of human data (Sendín et al., 2025). Future research should also consider the potential to collect AoA for data such as multi-word expressions, idioms, and other linguistic constructs of interest in behavioral research, but with limited AoA resources.

AI-generated estimates are valuable when stimuli need to be matched on a particular variable, but less acceptable when a variable is the focus of research. For such a variable, it is good to keep in mind Trott’s (2024) observation that the validity of LLM-generated norms depends upon a human gold standard with which to evaluate those norms (see also Conde et al., 2025). This is indeed why we invested resources in Study 1 to obtain human AoA ratings for reading and writing. The last thing we want is for human word recognition models to be based entirely on LLM estimates. When a variable is of fundamental theoretical importance, it should be investigated with human data. At the same time, LLM estimates can be used as supplementary and for finding interesting stimuli and for checking whether the human data is useful (for example, to check whether participants took a rating task in an online study seriously).

There are benefits of using LLMs to collect psycholinguistic data, as this study and others show, but significant issues must be acknowledged. As illustrated in the discussion of Chain-of-Thought (CoT) aside of Study 3, a word like ‘colicky’ is likely learned in highly context-dependent ways (e.g., earlier by older siblings in a family with a baby, but later and often in adulthood by others). Contextual factors, cultural variations, and personal experiences heavily influence when words are acquired, meaning AoA norms derived from LLMs represent only typical patterns and should not be treated as normative for all learners. Furthermore, the stability of LLM-generated ratings across model updates and between different LLMs needs more investigation. The studies reported did not compare models, relying solely on GPT-4, which is reasonable as it showed the highest correlations with human benchmarks across a number of variables (Conde et al., 2025). Still, we would hesitate to recommend a set of LLM-generated estimates as psycholinguistic norms without some basic validation checking against human ratings. Indeed, Conde et al. (2025) found substantial differences across the models and variables tested. Such studies remind us that human-validated data is critical for ongoing theoretical research.

## Availability

**Fig. 9:**
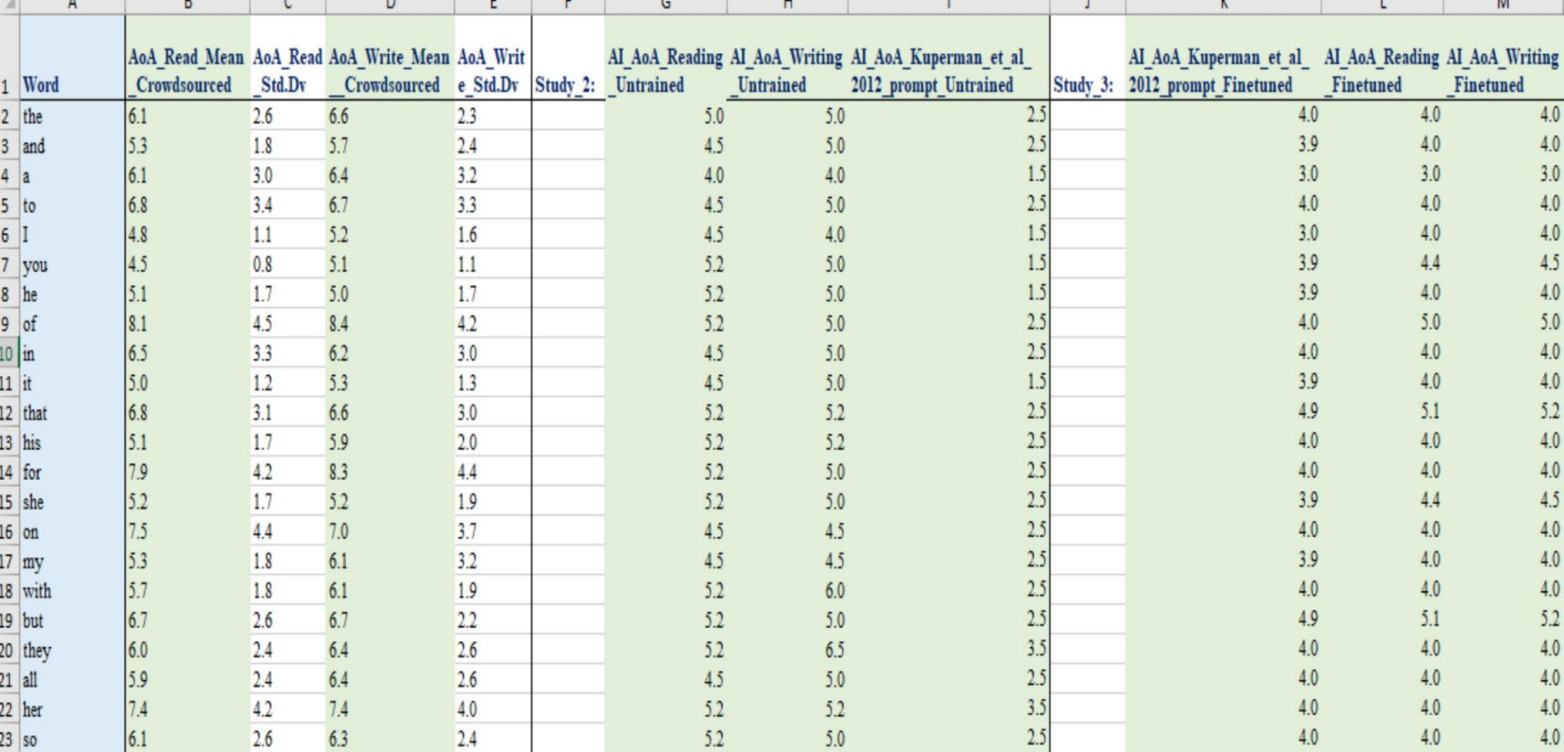
Crowdsourced print norms from Study 1 + AI Supplements (Study 1 and 3)

All resources are available in the OSF: https://tinyurl.com/3ck778nr. Study 1 and Study 2 AoA estimates are combined in a spreadsheet as shown in a sample provided in Fig. 9. The AoA_Read_Mean_Crowdscourced and AoA_Write_Mean_Crowdscourced columns are the crowdsourced average estimates and are the most important, while the untrained AI results from Study 2 are appended for reference in the right columns followed by the fine-tuned models of Study 3.

**Fig. 10:**
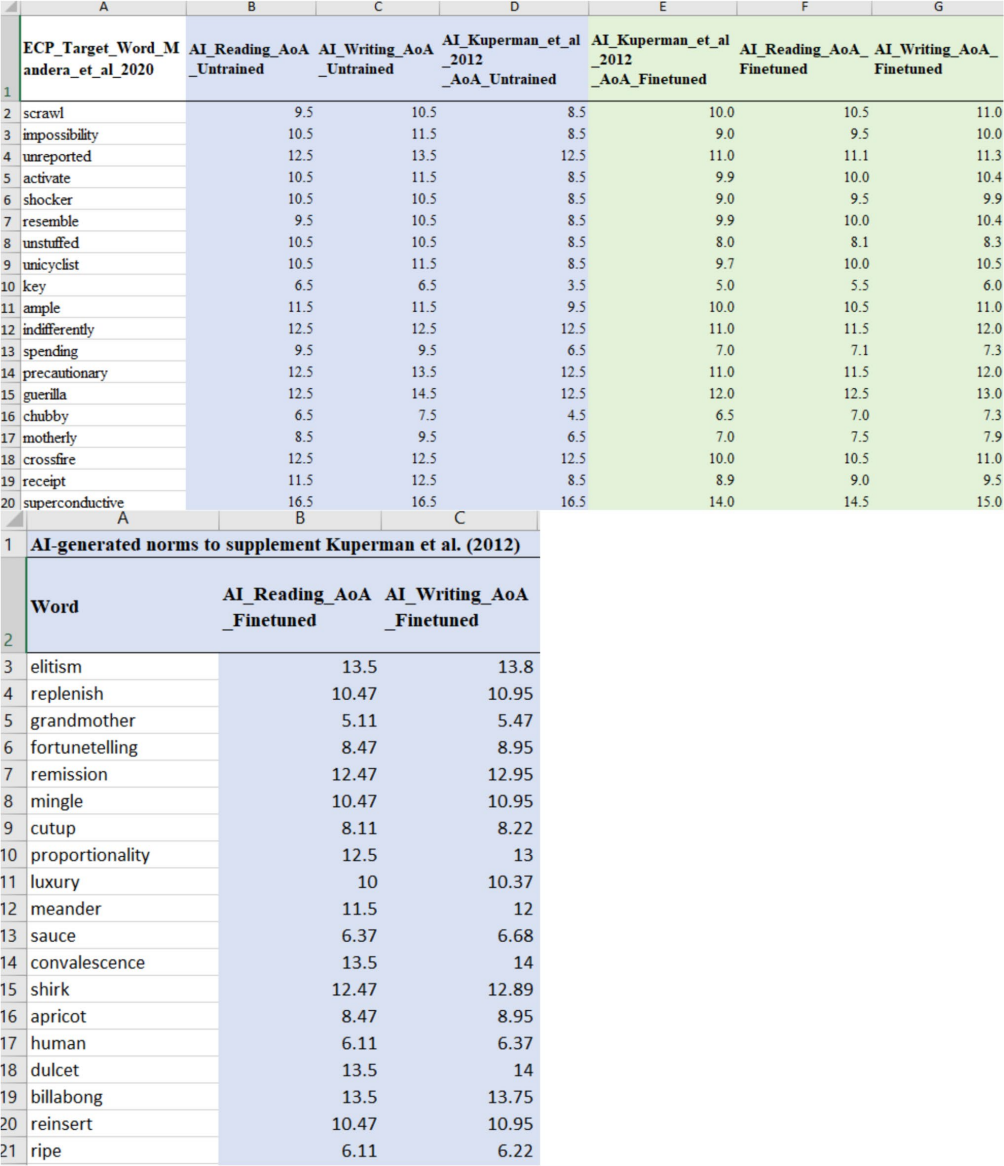
AI-generated AoA estimates for the ECP and Kuperman et al. (2012)

The results from Study 3 words are provided in two separate spreadsheets, one that supplements the ECP and one that supplements Kuperman et al. (2012). Figure 10 shows the format of these AI-generated AoA norms. They are on separate spreadsheets freely available in the OSF. The ECP example in Fig. 10 shows columns that represent the output from the three different prompts given to the AI. The first three columns are the print-related prompts and the Kuperman et al. (2012) prompt before model training; the last three give the information after training.

The availability of different AI-generated estimates allows users to assess how stable the estimates for a particular word are. Large differences indicate uncertainty, which can be problematic in some studies and, in others, the start of an interesting new line of research.

## Limitations

There are several limitations in any study. Though at each step due diligence was undertaken to produce norms of value, the norms generated for the community from these investigations are not without flaws.

With regard to Study 1, the results showed that the new crowdsourced reading and writing estimates tend to be highly correlated with each other, so may represent the same thing for participants. This may also reflect an order effect of the instrument, with reading estimates provided before writing estimates, with participants perhaps consistently rating writing AoA slightly higher than their reading estimate. In addition, there is likely a level of noise in the crowdsourced data, reducing quality. While we screened all data through extensive quality checks, there is no guarantee that the workers in AMT always provided their best possible answers. The AMT platform has challenges, such as worker inattentiveness, self-misrepresentation, high attrition and the existence of web-robots (bots), all of which can affect data quality (Aguinis et al., 2021).

As with other norms, some AoAs for certain words seem unlikely, so researchers must apply critical judgment when they use data (e.g., by also considering the zero-shot AI-generated estimates for those words). Note also that words can have multiple meanings, and we have not explored this aspect here. We do not know which meaning was in mind for polysemous words that were being rated in any of the three studies. This is not a limitation specific to this study, but is important as word meanings are not all acquired at the same age. The CoT discussion above points to interesting future research on polysemy using AI as a tool to disambiguate meanings and estimate separate AoAs for different senses.

AI limitations include that it sometimes misses words and produces incorrect output. These were corrected where identified, but there may still be errors in the databases, given the number of words that have been analyzed. The AI also refused to give estimates for taboo words. Additionally, the ‘black box’ problem is that we do not have a clear understanding of the data used by the LLM to predict word characteristics. Finally, since LLMs are in constant development, one challenge for the new wave of AI research is the potential that AI data-generated word norms may have a short lifespan as the performance of predictive AI systems improves over time. This may limit the timeframe at which the AI resources of Study 3 are going to be of value to the community. However, updated AI models may not necessarily improve AoA for some time, given that this is not a particular focus of the LLMs training, so estimates in the resources may remain consistent for years to come. In addition, the high correlations with human data in this study indicate that the gain expected would be minimal.

## Conclusion

This paper has released multiple new AoA databases that build upon the most widely used AoA norms of Kuperman et al. (2012). The first contribution was a crowdsourced megastudy that supplements the Kuperman et al. (2012) norms, providing estimates of the reading and writing ages for the early acquired words in that database. This subset of words needed to be reassessed because the original prompt tapped oral language comprehension, which is typically earlier than print, as reflected by the new print estimates. The second study investigated how well these crowdsourced norms could be replicated by the LLM GPT-4. It was found that they replicated human ratings well, and performed well in predicting word processing efficiency. This finding provided Study 3 with confidence to develop AoA estimates that can be extended to larger existing databases (Brysbaert et al., 2019; Mandera et al., 2020). This paper has demonstrated that future research should be able to obtain reasonably good AoA estimates for any words not provided in the newly released databases of this study and previous studies, and added to the growing consensus around the value of AI for supplementing, or functioning in lieu of, crowdsourcing megastudies.

## Data Availability

All data is available in the OSF: https://tinyurl.com/3ck778nr

## Code availability

Open Science Framework: https://tinyurl.com/3ck778nr.

## Declarations

### Open practices statement

All data, code and resources are available in the OSF: https://tinyurl.com/3ck778nr.
